# The intergenerational relationship between conditional cash transfers and newborn health

**DOI:** 10.1186/s12889-022-12565-7

**Published:** 2022-01-30

**Authors:** Andreza Daniela Pontes Lucas, Monaliza de Oliveira Ferreira, Tarcisio Daniel Pontes Lucas, Paola Salari

**Affiliations:** 1 Federal University of Pernambuco, Caruaru, Pernambuco Brazil; 2 Catholic University of Pernambuco, Pernambuco, Brazil; 3 Swiss Tropical and Public Health Institute, Basel, Switzerland; 4 Institute of Pharmaceutical Medicine (ECPM), University of Basel, Basel, Switzerland

**Keywords:** Conditional cash transfer programs (CCT), *Bolsa Familia*, Intergenerational health, Child health, Maternal health

## Abstract

**Background:**

Lack of nutrition, inadequate housing, low education and limited access to quality care can negatively affect children’s health over their lifetime. Implemented in 2003, the Bolsa Familia (“Family Stipend”) Program (PBF) is a conditional cash transfer program targeting poor households in Brazil. This study investigates the long-term benefits of cash transfers through intergenerational transmission of health and poverty by assessing the early life exposure of the mother to the PBF.

**Methods:**

We used data from the 100M SINASC-SIM cohort compiled and managed by the Center for Data and Knowledge Integration for Health (CIDACS), containing information about participation in the PBF and socioeconomic and health indicators. We analyzed five measures of newborn health: low (less than 2,500 g) and very low (less than 1,500 g) birth weight, premature (less than 37 weeks of gestation) and very premature (less than 28 weeks of gestation) birth, and the presence of some type of malformation (according to ICD-10 codes). Furthermore, we measured the early life exposure to the PBF of the mother as PBF coverage in the previous decade in the city where the mother was born. We applied multilevel logistic regression models to assess the associations between birth outcomes and PBF exposures.

**Results:**

Results showed that children born in a household where the mother received BF were less likely to have low birth weight (OR 0.93, CI; 0.92-0.94), very low birth weight (0.87, CI; 0.84-0.89), as well as to be born after 37 weeks of gestation (OR 0.98, CI; 0.97-0.99) or 28 weeks of gestation (OR 0.93, CI; 0.88-0.97). There were no significant associations between households where the mother received BF and congenital malformation.

On average, the higher the early life exposure to the PBF of the mother, the lower was the prevalence of low birth weight, very low birth weight and congenital malformation of the newborn. No trend was noted for preterm birth.

**Conclusion:**

The PBF might have indirect intergenerational effects on children’s health. These results provide important implications for policymakers who have to decide how to effectively allocate resources to improve child health.

**Supplementary Information:**

The online version contains supplementary material available at 10.1186/s12889-022-12565-7.

## Background

Poor health is very often associated with lack of economic resources. Due to poor nutrition, substandard housing, low education and scarce or inefficient access to healthcare, being born in a family suffering from economic conditions can negatively affect children’s health over their entire lives. The literature highlights the intergenerational transmission of health and how this might result in a self- perpetuated poverty (and poor health). One study estimated that about one-third of children’s health status is affected by parents’ health [1], and another study also indicated the transmission of health inequality among generations [2]. Still other studies have shown that health status of the mother over her entire lifetime matters for the health status of the subsequent generation [3–5].

Cash transfers targeted to poor households have often been used as a strategy to break this vicious circle and to increase human capital [6]. If conditions are attached to the transfer, cash transfers can improve both income and behavioral outcomes linked to health and education [7–13]. There is an increasing number of studies highlighting how cash transfers may be effective in decreasing poverty while also reducing inequality and promoting economic growth [14, 15].

This paper investigates the relationship between a conditional cash transfer designed to improve health and education of children in Brazil, and health achievements of newborns in the next generation.

Implemented in 2003, the *Bolsa Familia* (“Family Stipend”) Program (hereinafter PBF) is a conditional cash transfer program targeting poor and extremely poor households in Brazil. Poor households are eligible for the program if their per capita income is between BRL 89.01 and 178.00 (about USD 17 and 34, threshold values of 2020) and one member is a pregnant woman (or a woman who just gave birth), or a child up to 17 years old. Households with per capita income up to BRL 89, are considered to be extremely poor and are eligible independent of their composition [7, 16]. However, receipt of PBF assistance does not depend only on the eligibility criteria, but also on the program’s budget and political decisions [7, 16]. In 2020, the number of households receiving *Bolsa Familia* (BF) aid was more than 13.9 million [16].

The main goals of the PBF are three: reduction of poverty and inequality; increased human capital; and social integration and empowerment of PBF participants, who are connected to services like social assistance programs and employment training [17]. The conditions for a household to get the subsidy include: vaccines for children under seven; medical checkup for woman from 14 to 44; prenatal visits for pregnant woman, medical checkups for breast feeding woman and their children; a minimum of 85% school attendance for children from 6 to 15 years old; and at least 75% school attendance for 16- and 17-year olds [8].

There is extensive literature on the PBF and its association with socioeconomic indicators, such as poverty and inequality reduction [15, 18], better school results and higher school attendance [19, 20] and higher social security coverage, due to labor formalization, and labor income [21]. Moreover, the PBF contributes to decrease the gender gap in two ways. It enhances female empowerment, since the woman is generally responsible for receiving the transfer [22]; and it increases school participations of girls, especially in rural and remote areas [20].

Despite wide research on the effects of the BFP on children’s health, there is no evidence of the potential intergenerational effects of this program on health. Some evidence has been reported of the potential long-term positive effects on education and exit from poverty [23, 24].

In this study, we consider three health measures that have been shown to be correlated with mothers’ socioeconomic condition and at the same time to have potentially life-long health effects on the child, namely: low birth weight; preterm birth and congenital malformation. While there is wide consensus that socioeconomic conditions of the mother play a role in reducing the probability of low birth weight [25–35], and preterm births [25, 26, 30, 35–37], the association with congenital malformation is weaker and ambiguous [38–45].

Effects of low birth weight are also linked with other life-long health effects of the child. Despite some weak evidence [46, 47], the majority of studies report a positive association between birth weight and intelligence [48, 49] and language development of children [50]. Evidence of inverse correlation of birth weight and risk of metabolic syndrome [51] and diabetes prevalence [52] in adults has also been found. Similarly, preterm birth was found to be associated with high adult blood pressure [53]; poor growth, hospital admissions, longstanding illness/disability, asthma [54]; motor and psychological development and well-being during the entire life [55]; language development [50]; higher risk of physical and behavioral health problems [56] and even low intelligence quotient and autism spectrum disorder [55]. Finally, literature on the effects of severe congenital malformation on adult daily life, especially if combined with other health problems [57, 58], also exists.

This study aims to assess the long-term benefits of cash transfers through intergenerational transmission of health and poverty, by assessing the relationship between BF aid received by the mother during childhood and newborn health, controlling for a set of socioeconomic and health variables. In other words, we investigate the probability that a grandmother’s receipt of BF aid has brought some positive effects not only on her daughter but also on her grandchild.

To do so, we used national level data including all children born from 2011 to 2015 from households enrolled in social assistance programs for which information on birth and mothers’ background was available (more than 5 million children).

We applied multilevel logistic regression models with municipal random effects to assess the correlations of birth outcomes, conditional cash transfers, socioeconomic and health variables.

Section 2 shows the data used in the analysis; section 3 presents the methods used in the study; section 4 reports and analyzes the results; section 5 discusses the results; and section 6 concludes.

## Methods

### Data

We used data from the 100M SINASC-SIM cohort compiled and maintained by the Center for Data and Knowledge Integration for Health (CIDACS) [59]. The cohort data files link anonymized information from three national datasets: the *Cadastro Unico* (CADU), the Newborn Information System (SINASC) and the Mortality Information System (SIM). CADU [60] is a census dataset created by the Brazilian government aiming to improve the targeting of social programs (e.g., PBF) and collecting information on all people benefitting from at least one of the social programs organized by the government. The CADU database includes individuals from households eligible for the federal social programs. This means that all individuals in our database were part of one or more social inclusion programs. The CADU database does not track individuals over time, but rather shows the most recent information for each variable. The SINASC [61] covers all children born alive in Brazil. It includes information on prenatal care, birth outcomes and mother’s social indicators. Finally, the SIM [61] is a dataset containing information on deaths. The cohort 100M SINASC-SIM includes 114,001,661 million individuals from 40,542,929 households. We used a subsample of mothers and children from the cohort 100M SINASC-SIM, excluding the observations with no SINASC information. This resulted in a dataset with 31,331,817 mothers or children. We reshaped the database in order to match each child with his or her mother’s information, thus identifying 16,448,931 child-mother pairs. Among these pairs, we only kept those where the child was born from 2011 to 2015 (last year available), namely 5,246,874 individuals. The choice of the initial year (2011) was due to the method used by CIDACS to build the 100M SINASC-SIM cohort. Starting from 2011, the individual information of the CADU and SINASC datasets was merged by matching the name and the whole date of birth (day, month and year) of people [62, 63]. Before 2011, only the name and the year of birth was considered, which might have led to less data accuracy. Moreover, we added to our database other variables at the municipal level. We included the number of households benefitting from the BFP over the total population in each municipality and each year, which we used as a proxy for mother exposure to the BFP from the beginning of the program up to 2010. Finally, we added municipal GDP, municipal population and poverty index for the years considered. GDP and population data were taken from the *Brazilian Institute of Geography and Statistics* (IBGE, [64]), BF indicators from the Brazilian Open Data Portal [65] and poverty data from the Atlas of Human Development in Brazil [66].

### Empirical strategy

We employed multilevel logistic regression models with municipality random effects to assess the associations between health outcomes at birth and conditional cash transfers, controlling for socioeconomic and health variables of the mother. We aimed to assess the effects of both BF transfers: the one received during pregnancy, and the one potentially received when the mother was a child. The dependent variables were dichotomous variables equal to one if the child suffered from a negative health outcome, and zero otherwise. We also controlled for year fixed effects.

Three health outcomes at birth were considered as objective measures of newborns’ health: birth weight, preterm birth and presence of some type of malformation. We considered two levels of birth weight: low (less than 2500 g), very low (less than 1500 g). We analyzed two levels of preterm birth: premature (less than 37 gestation weeks) and very premature (less than 28 gestation weeks). Finally, we considered the presence of any type of malformation according to the International Classification of Diseases (ICD-10) codes [67].

### Missing data

The percentage of observations with at least one missing value in one of the variables was 45%. To deal with this missing data issue, we assumed a missing at random (MAR) pattern of missing values and performed a statistical MI based on the chained equation method, using the *mi impute* command in Stata 15 [68, 69].

### Primary and secondary exposure

Two variables represent BF subsidy: “*BF uptake” and* “*Prob early exposure”.” BF uptake”* is a dichotomous variable that assumes value one if the mother received BF benefits during pregnancy, and zero otherwise. In order to assess the intergenerational impact of the conditional cash transfer and avoid biased results, we had to use a proxy due to data limitations. In fact, it was possible to individually link each mother with her mother (i.e., the grandmother) only for a very restricted number of generally very young mothers (less than 0.1% of the total sample). Therefore, we created a municipal level proxy - “*Prob early exposure” -* to account for the probability that the mother benefitted from cash transfers during her childhood.

This variable was computed as the ratio of the number of families benefitting from the BFP over the total population in each municipality and each year, averaged for the number of years considered (2004-2010). An underlying assumption is that the mother did not change residence municipality during her youth.

Since we consider children born from 2011 to 2015, we used the BF prevalence in the previous decade to estimate the probability of the mother’s being a BF beneficiary during her childhood and adolescence. We split the “*Prob early exposure”* index into five ordered categories with equal density (from very low to very high prevalence).

To control for the potential confounding effects of other programs aimed at reducing poverty, we added a variable to estimate the reduction of poverty in the decade 2000-2010 (*Poverty reduction*). “*Prob early exposure”* and “*Poverty reduction”* were the only variables constant over time in the model.

Due to poor quality of income data, we estimated an asset-wealth-index as a proxy for income [70]. The index was created using principal component analysis, based on the following household characteristics: electrification; municipal water supply; presence of an indoor bathroom; municipal garbage collection; sewage system; type of house; and building material. Households were ranked into quintiles, from richest to poorest. We also included municipal GDP per capita and year fixed effects as additional controls.

### Other variables

We added the following social indicators in the model: mother’s education; marital status; age; child race and gender; and residence in a rural or urban area. Five levels of education were considered: “*No education*” if the mother never attended school, “*Literacy*” if the mother had learned to read, “*Until 5*^*th*^ *grade*” if the mother had accomplished the first half of primary education, “*Until 9*^*th*^ *grade*” if the mother had finished primary education, and “*Secondary or more*” if mother had secondary school diploma or higher degree. We classified mother’s age in four groups: from 10 to 19 years old; from 20 to 29 years old; from 30 to 39 years old; and 40 years old or more. Marital status was divided in four different groups: single; married; widowed; or divorced. Ethnicity of the child was divided into: “white”; “yellow”; “black”; “brown”; or “indigenous”.

We controlled for cases of twins or triplets (*Multiple pregnancy*). We included information on mother’s health, controlling whether she had a previous childbirth (*First gestation*), and if so, whether she had a previous loss (*Previous fetal loss)*. Finally, the variable “*Place of birth*” indicated whether the delivery occurred in a hospital, other healthcare facility, at home or in another place.

## Results

Table 1 shows descriptive statistics (number of observations, mean, min and max) for all the variables used in the analysis. Additional descriptive statistics can be found in the Supplementary material (Tables S1 and S2). In particular, given that our analyses are based on a sample of vulnerable people (i.e., individuals who were part of at least one social inclusion program), we compared the prevalence of each health outcome and other variables with the values of the whole Brazilian population (see Table S1). Results showed that in our sample there were slightly more single, teenage and black mothers than in the total Brazilian population. Child health outcome problems were slightly less that the Brazilian average. This might indicate that some children with health problems did not survive long enough to be registered in the database.

**Table 1:** Descriptive statistics of our database (from the 100M SINASC-SIM cohort)

Variable	N. (Observations)	Mean	Min	Max
**Child health outcomes**				
LBW	5,242,209	0.073	0	1
VLBW	5,242,209	0.007	0	1
PTB	5,019,754	0.108	0	1
VPTB	5,019,754	0.002	0	1
CFM	5,246,673	0.006	0	1
**BF exposure**				
BF uptake	5,246,798	0.233	0	1
Prob early exposure				
Q1 (Lowest)	5,245,137	0.2	0	1
Q2	5,245,137	0.2	0	1
Q3	5,245,137	0.2	0	1
Q4	5,245,137	0.2	0	1
Q5 (Highest)	5,245,137	0.2	0	1
**Economic indicators**				
Poverty reduction	5,245,905	15.132	-22.08	76.91
GDP per capita	5,245,764	26.567	0	1,030.184
Wealth Index				
Q1 (Richest)	4,410,368	0.17	0	1
Q2	4,410,368	0.21	0	1
Q3	4,410,368	0.207	0	1
Q4	4,410,368	0.213	0	1
Q5 (Poorest)	4,410,368	0.2	0	1
**Social indicators**				
Mother’s education				
No education	4,557,291	0.046	0	1
Literacy	4,557,291	0.008	0	1
Until 5th grade	4,557,291	0.283	0	1
Until 9th grade	4,557,291	0.367	0	1
Secondary or more	4,557,291	0.296	0	1
Mother’s age group				
10-19	5,245,195	0.242	0	1
20-29	5,245,195	0.498	0	1
29-39	5,245,195	0.245	0	1
40 plus	5,245,195	0.024	0	1
Marital status				
Single	5,170,896	0.484	0	1
Married	5,170,896	0.506	0	1
Widowed	5,170,896	0.002	0	1
Divorced	5,170,896	0.008	0	1
Female	5,246,673	0.489	0	1
Race/color				
White	5,246,673	0.328	0	1
Black	5,246,673	0.035	0	1
Yellow	5,246,673	0.006	0	1
Brown	5,246,673	0.622	0	1
Indigenous	5,246,673	0.009	0	1
Rural	5,246,118	0.216	0	1
**Health indicators**				
Pregnancy				
Single	5,235,314	0.986	0	1
Double	5,235,314	0.014	0	1
Triple	5,235,314	0	0	1
First pregnancy	4,503,804	0.306		
Previous fetal loss	4,655,849	0.195		
Place of birth				
Hospital	5,245,547	0.98	0	1
Other health establishment	5,245,547	0.009	0	1
Home	5,245,547	0.009	0	1
Other	5,245,547	0.002	0	1
N	5,246,874			

*Notes*: *LBW* low birth weight, *VLBW* very low birth weight, *PTB* preterm birth, *VPTB* very preterm birth, *CFM* congenital malformations. “Prob early exposure” indicates the probability of social protection exposure during mother’s childhood

Results from the multilevel analysis are presented in Table 2. Children born in households where the mother received BF aid were less likely to have low birth weight (hereinafter LBW) (OR 0.932, 95% CI; 0.924-0.941), very low birth weight (hereinafter VLBW) (OR 0.872, 95% CI; 0.848-0.897), as well as to be born after 37 weeks of gestation (hereinafter PTB) (OR 0.979, CI; 0.971-0.986) or 28 weeks of gestation (hereinafter VPTB) (OR 0.903, CI; 0.887-0.975). There was no significant effect on congenital malformation (hereinafter CMF).

**Table 2:** Results of the logistic multilevel regression estimates

	LBW	VLBW	PTB	VPTB	CMF
**BF Exposure**					
BF uptake	0.932***	0.872***	0.979***	0.930**	1.012
	[0.924-0.941]	[0.848-0.897]	[0.971-0.986]	[0.887-0.975]	[0.982-1.042]
Prob early exposure					
Q1 (Lowest)	(reference)	(reference)	(reference)	(reference)	(reference)
Q2	0.919***	0.849***	0.936***	0.892*	0.810***
	[0.894-0.944]	[0.799-0.903]	[0.910-0.962]	[0.807-0.985]	[0.747-0.879]
Q3	0.831***	0.738***	0.922***	0.885*	0.716***
	[0.807-0.854]	[0.691-0.790]	[0.897-0.949]	[0.795-0.986]	[0.657-0.780]
Q4	0.741***	0.695***	0.932***	0.92	0.686***
	[0.719-0.765]	[0.645-0.749]	[0.904-0.961]	[0.814-1.034]	[0.623-0.754]
Q5 (Highest)	0.690***	0.707***	0.946***	0.956	0.617***
	[0.668-0.713]	[0.653-0.765]	[0.916-0.976]	[0.843-1.084]	[0.558-0.681]
**Economic indicators**					
Wealth Index					
Q1 (Richest)	(reference)	(reference)	(reference)	(reference)	(reference)
Q2	1.005	0.947**	0.996	0.946	0.979
	[0.993-1.017]	[0.913-0.982]	[0.986-1.007]	[0.884-1.011]	[0.943-1.016]
Q3	0.993	0.936**	0.979**	0.927*	0.956*
	[0.980-1.005]	[0.898-0.975]	[0.967-0.990]	[0.872-0.986]	[0.917-0.996]
Q4	0.995	0.900***	0.980**	0.879**	0.978
	[0.982-1.009]	[0.866-0.935]	[0.969-0.991]	[0.816-0.947]	[0.940-1.019]
Q5 (Poorest)	0.996	0.860***	0.986*	0.873**	0.947*
	[0.982-1.010]	[0.824-0.897]	[0.974-0.998]	[0.803-0.949]	[0.904-0.993]
**Social indicators**					
Mother's education					
No education	(reference)	(reference)	(reference)	(reference)	(reference)
Literacy	0.947*	1.053	0.957*	1.098	0.898
	[0.902-0.994]	[0.902-1.229]	[0.925-0.991]	[0.903-1.334]	[0.756-1.068]
Until 5th grade	0.946***	0.955	0.951***	0.921	0.979
	[0.929-0.963]	[0.905-1.007]	[0.933-0.969]	[0.839-1.010]	[0.921-1.040]
Until 9th grade	0.882***	0.968	0.860***	0.828***	0.972
	[0.867-0.899]	[0.916-1.023]	[0.845-0.875]	[0.757-0.906]	[0.916-1.031]
Secondary or more	0.830***	1.021	0.819***	0.821***	0.97
	[0.814-0.847]	[0.961-1.084]	[0.804-0.835]	[0.751-0.898]	[0.911-1.032]
Mother's age group					
10-19	(reference)	(reference)	(reference)	(reference)	(reference)
20-29	0.900***	0.936***	0.776***	0.682***	0.999
	[0.891-0.910]	[0.906-0.967]	[0.769-0.783]	[0.649-0.717]	[0.965-1.033]
29-39	1.071***	1.268***	0.853***	0.706***	1.122***
	[1.059-1.084]	[1.221-1.317]	[0.843-0.862]	[0.664-0.751]	[1.079-1.168]
40 plus	1.515***	1.745***	1.121***	0.948	1.724***
	[1.483-1.547]	[1.640-1.856]	[1.101-1.142]	[0.850-1.058]	[1.616-1.839]
Marital status					
Single	(reference)	(reference)	(reference)	(reference)	(reference)
Married	0.934***	0.955***	0.970***	0.795***	1.016
	[0.927-0.941]	[0.935-0.977]	[0.964-0.976]	[0.765-0.826]	[0.992-1.041]
Widowed	0.975	0.897	0.992	0.991	1.334*
	[0.900-1.056]	[0.704-1.144]	[0.928-1.060]	[0.656-1.497]	[1.068-1.666]
Divorced	0.997	1.007	1.027	0.84	1.119*
	[0.961-1.034]	[0.907-1.117]	[0.995-1.060]	[0.677-1.043]	[1.003-1.249]
Female	1.282***	1.157***	0.936***	1.033	0.730***
	[1.274-1.291]	[1.134-1.182]	[0.931-0.942]	[0.994-1.073]	[0.714-0.747]
Race/color					
White	(reference)	(reference)	(reference)	(reference)	(reference)
Black	1.186***	1.175***	1.116***	1.316***	0.985
	[1.165-1.208]	[1.114-1.240]	[1.099-1.134]	[1.198-1.445]	[0.928-1.047]
Yellow	1.077**	1.137	1.048*	0.96	1.024
	[1.029-1.128]	[0.994-1.302]	[1.009-1.088]	[0.745-1.237]	[0.882-1.189]
Brown	1.065***	1.037**	1.055***	1.135***	0.934***
	[1.056-1.074]	[1.012-1.063]	[1.047-1.062]	[1.085-1.187]	[0.910-0.959]
Indigenous	0.968	0.715***	1.347***	0.831	0.907
	[0.923-1.014]	[0.611-0.837]	[1.303-1.393]	[0.653-1.058]	[0.774-1.063]
Rural	0.96	0.973	0.962***	0.965	1.014
	[0.949-0.971]	[0.939-1.008]	[0.953-0.971]	[0.902-1.031]	[0.975-1.054]
**Health indicators**					
Pregnancy					
Single	(reference)	(reference)	(reference)	(reference)	(reference)
Double	17.763***	9.626***	8.262***	5.781***	1.257***
	[17.497-18.032]	[9.315-9.948]	[8.136-8.390]	[5.390-6.199]	[1.158-1.365]
Triple	63.925***	52.974***	31.134***	19.187***	1.188
	[55.563-73.544]	[46.631-60.181]	[27.280-35.532]	[14.457-25.466]	[0.637-2.215]
First pregnancy	1.581***	1.652***	1.041***	1.056*	1.230***
	[1.565-1.596]	[1.601-1.704]	[1.031-1.051]	[1.006-1.109]	[1.194-1.267]
Previous fetal loss	1.371***	1.629***	1.120***	1.270***	1.155***
	[1.358-1.384]	[1.585-1.675]	[1.110-1.130]	[1.211-1.332]	[1.120-1.192]
Place of birth					
Hospital	(reference)	(reference)	(reference)	(reference)	(reference)
Other health establishment	0.672***	0.542***	0.861***	0.958	1.11
	[0.641-0.704]	[0.460-0.639]	[0.829-0.894]	[0.782-1.176]	[0.974-1.266]
Home	1.686***	2.274***	1.777***	2.795***	0.846*
	[1.628-1.746]	[2.055-2.515]	[1.723-1.833]	[2.377-3.287]	[0.727-0.984]
Other	3.317***	3.798***	2.210***	3.822***	1.422**
	[3.115-3.533]	[3.242-4.449]	[2.092-2.335]	[2.987-4.892]	[1.104-1.828]
N	5246874	5246874	5246874	5246874	5246874

*Notes*: *LBW* low birth weight, *VLBW* very low birth weight, *PTB* preterm birth, *VPTB* very preterm birth, *CFM* congenital malformations. “Prob early exposure” indicates the probability of social protection exposure during mother’s childhood. Year fixed effects included in the estimate. Indicators of poverty reduction and GDP per capita at municipal level are included in the model. Results presented in odds ratios; 95% confidence intervals in brackets. *P<0.05; **P<0.01; ***P<0.001

Results of the proxy for intergenerational effect of the BFP suggested that the highest probability that the grandmother received high levels of social support during childhood (measured as the prevalence of BFP in the decade before child’s birth in a given municipality), was associated with the lowest probability of LBW (OR=0.690, 95% CI: 0.668-0.713), VLBW (OR=0.707, 95% CI: 0.653-0.765), and CMF (OR=0.617, 95% CI: 0.558-0.681) of the newborn. The highest probability that the grandmother received BF was also correlated with a lower probability of PTB, but with a coefficient closer to one (OR=0.946, 95% CI: 0.916-0.976). These findings suggest that in the municipalities where it was implemented, the BFP contributed to reduce health problems in the two subsequent generations.

The variable “*Poverty reduction*”, a control for other municipal-level antipoverty policies in the decade 2000-2010, was equal to 1 for LBW and not significant for the other health outcomes. This reinforces the main results, suggesting that the presence of the PBF at the time when the mother was a child was more effective in improving birth outcomes than other antipoverty policies. The same holds for municipal-level per-capita GDP: it showed no significant effect on all the birth outcomes considered.

Wealth was correlated with the health outcomes. Children born in the poorest households had slightly lower probability of suffering from VLBW (OR= 0.860, 95%CI: 0.824-0.897), PTB (OR= 0.986, 95%CI: 0.974-0.998), VPTB (OR= 0.873, 95%CI: 0.803-0.949) and CMF (OR= 0.947, 95%CI: 0.904-0.993) than those born in the richest households. Education showed a gradient effect: LBW (OR= 0.830, 95%CI: 0.814-0.847), PTB (OR=0.819, 95% CI: 0.804-0.835) and VPTB (OR=0.821, 95% CI: 0.751-0.898) were less likely among more educated mothers. There was no significant correlation between mother’s education and either VLBW or CMF.

Results of age showed that mothers from 20 to 29 years old were associated with the healthiest children in all models (LBW: OR= 0.900, 95% CI: 0.891-0.910; VLBW: OR= 0.936, 95% CI: 0.906-0.967; PTB: OR=0.776, 95% CI: 0.769-0.783; VPTB: OR=0.682, 95% CI: 0.649-0.717). In contrast, mothers over 40 were more likely to have children with LBW (OR=1.515, 95% CI: 1.483-1.547), VLBW (OR=1.745, 95% CI: 1.640-1.856), PTB (OR=1.121, 95% CI: 1.101-1.142), and CMF problems (OR=1.724, 95% CI: 1.616-1.839) than very young mothers (10-19 years old).

Marital status was also significantly correlated with children’s health outcomes. Married women showed the lowest probability of having children with LBW (OR=0.934, 95% CI: 0.927-0.941), VLBW (OR=0.955, 95% CI: 0.935-0.977), PTB (OR=0.970, 95% CI: 0.964-0.976) and VPTB (OR=0.795, 95% CI: 0.765-0.826) than single, widowed or divorced women.

Among children’s characteristics, LBW and VLBW occurred more likely among female children (OR=1.282, 95% CI: 1.274-1.291 and OR=1.157, 95% CI: 1.134-1.182, respectively), while PTB and CMF were less likely (OR=0.936, 95% CI: 0.931-0.942 and OR=0.730, 95% CI: 0.714-0.747, respectively). Black and brown children were more likely to suffer from LBW, VLBW, PTB and VPTB than white children.

Living in rural versus urban areas did not have a significant correlation with children’s health outcomes, with the only exception being a negative association between rural residence and probability of PTB (OR=0.962, 95% CI: 0.953-0.971).

Finally, all health problems considered were more likely to occur in the case of multiple pregnancies (twins and triplets) rather than single pregnancy, or if the mother was in her first pregnancy or if her previous pregnancy resulted in fetal loss. Moreover, children born in hospitals or in other health facilities were less likely to present LBW, VLBW, PTB and VPTB than those born at home or in other places.

## Discussion

In this study, we aimed to assess the intergenerational relationship between a conditional cash transfer program and newborn health, where intergenerational means three generations: from the grandmother to her daughter (i.e., the mother) and from the mother to the newborn. A previous study assessed the long-term effects of the BFP on education [24], but to the best of our knowledge this is the first study addressing the long-term effects on children’s health. The poor data quality relative to the grandmothers present in the 100M SINASC-SIM database, did not allow us to rely on their individual information. We therefore built a proxy to measure the likelihood that the mother was exposed to BF when she was a child, based on the prevalence of the BFP in the geographic area (municipality) at each point in time.

Furthermore, our analyses were based on data from two national databases (SINASC and CADU), which have been linked together only recently and have not yet been widely analyzed in the literature. In Brazil, the majority of studies merging both socioeconomic variables with birth outcomes were only conducted at the municipal or regional level. National datasets on health, despite having open access, do not normally provide economic information. Brazilian studies mostly refer only to one (or more) specific health facilities, as it is generally easier to collect data and merge economic and health information at such a small geographical level [37, 38, 71].

Our main findings suggest the presence of a correlation between the early-life exposure to the BFP of mothers and the health of their newborns. At the same time, the obtainment of BF benefits when the mother was pregnant or had children showed a positive correlation with the health of the newborn, in line with what is generally found in the literature [13, 19, 22, 72–74]. Therefore, not only did the aid received by the mother have a positive effect on the health outcomes of the baby, but receipt by the grandmother also increased the probability of better health of her newborn grandchild.

From our analysis, it was not possible to disentangle the reasons why the BFP is associated with better child health outcomes: it is likely that women from households receiving BFP benefits received more adequate antenatal care, and this in turn is correlated with better child outcomes, as highlighted in many studies also focused on Brazil [37, 71, 75–78]. One study found that thanks to BF implementation, the average number of antenatal care visits increased from 3.5 in 2005 to 4.4 in 2009 [19]. Table S2 in the Supplementary material reports information about the average number of visits. From 2011 to 2015, 57.7% of the mothers paid more than 6 antenatal visits. To validate the hypothesis of correlation between more adequate antenatal care and better child outcome, we performed supplementary analysis adding the number of antenatal visits among the regressors. As expected, the results showed a significant, albeit smaller, effect of the BFP and a positive and significant effect of the number of antenatal care visits (Table S3 in the Supplementary materials). Hunter (2017), Glassman (2013) and Celhay (2017) underlined the positive relationship between conditional cash transfers and improved prenatal care [33, 79, 80].

Previous research also has associated the BFP with an increase in household’s food consumption [19, 22, 81].

Similarly, our results did not allow completely clarifying the reasons for the intergenerational beneficial effect of the BFP. Our hypothesis is that better health at birth and during childhood also meant better health as an adult and as a mother, as found in many studies [2, 3, 47–49]. Moreover, better health as a mother is likely to be correlated with better health of the newborn [1–5].

Results of the control variables were mostly as expected and in line with other studies. Previous analyses in Brazil have found low birth weight to be related to socioeconomic conditions [26, 82]; low mother education [75, 77, 78, 82]; being a single mother [75, 77, 78, 82]; and with mother’s age under 20 or over 34 [71, 75, 77, 78, 83].

The results relative to wealth suggest that a better child health outcome was correlated with lower income, which can be explained by the “low birth weight paradox” [84–86]. It is more likely that in the group of poorest mothers, fewer newborns survived with LBW (other conditions being equal). Thus, it is less likely that LBW children were born in the group of poor mothers because many children did not survive: healthier children from the poorest mothers were more likely to survive. Other studies have reported a positive association between low socioeconomic status and presence of congenital malformation, albeit small and not always significant [39–42, 44, 45].

Low birth weight and VLBW were also more frequent in female babies, as also reported in the literature [28, 30, 32]. The opposite is true for PTB and CMF.

Multiple pregnancy and previous loss were found in the literature to be sources of LBW, PTB and also malformations [37, 75, 87–90]. Our results corroborate this evidence (with weaker findings for malformations).

Generally, the choice of giving birth at home was positively correlated with a higher probability of child health problems. The only exception was congenital malformation (OR=0.846), probably because women who discovered during antenatal visits that their child might have some congenital malformation chose to give birth in a medical facility.

Giving birth in a health facility rather than hospital was associated with a lower probability of having a child with adverse health outcomes (OR= 0.672 for LBW, OR=0.542 for VLBW and OR= 0.861 for PTB), while giving birth in another non-medical facility was associated with higher health risk of the child, probably due to emergency circumstances.

In general, the prevalence of VLBW and CMF in the newborn was less correlated with BF benefits. Both kinds of health problems were likely to be caused by factors whose control went beyond mothers’ choices and behavior. The general evidence of association of CMF with socioeconomic conditions was weaker than the other health outcomes. It is also possible that a subsidy such as BF might have had a role in improving the health of the child, but was more effective for less serious problems. When the adverse health condition of the child was too serious (as in the case of VLBW), the subsidy was not enough to prevent it.

Results on race suggested that children’s health outcomes were better for white children. This may have to do with the racial discrimination widespread in Brazilian society, since black and brown mothers generally have on lower income, lower educational level, and are more likely to be victims of violence [91].

This study has limitations, mainly due to constraints imposed by data availability. First, we could not link mothers with their mothers (i.e., babies with grandmothers), so we did not have individual data on the real obtainment of the BF aid of grandmothers. However, we created a proxy at the municipal level taking into account the eligibility of the women based on the prevalence of the BFP in any municipal area. Also, scant information was available on the health of the mother, an important determinant of the health of the child, in the dataset. Moreover, we considered women who were receiving BF aid as registered in the CADU database, but we did not have information on whether the mother was receiving the subsidy for the newborn with whom she was linked, or for another daughter or son. However, we are confident that this latter limitation did not bias our results, since the aid is generally beneficial for all household members. One more limitation is the fact that we did not consider babies born dead or who died shortly after birth, since they were not registered in the database. Furthermore, we could only look at mothers as main BF recipients, not fathers. However, CCT programs in Brazil are generally targeted to mothers [13, 22]. Finally, our analysis found statistically significant associations but the data did not allow assessing any causal pattern. More research would be needed to explore the long-term relationship of the BFP or similar programs, possibly also analyzing a causal relationship. Overall, this study showed promising potential long-term effects of the BFP.

## Conclusion

In this study we assessed the intergenerational relationship between the Brazilian *Bolsa Família* conditional cash transfer and newborns’ health over three generations. Using data from the cohort 100M SINASC-SIM we looked at the effects of the BFP from the grandmother to her daughter (i.e., the mother) and from the mother to the newborn. Our results suggest a potential positive intergenerational effect of the BFP. On average, the higher the probability that the grandmother received BF aid, the lower was the prevalence of low birth weight, very low birth weight and congenital malformation of the newborn. No trend was noted for preterm birth. At the same time, the obtainment of BF support by the mother showed a positive correlation with the health of the newborn.

These results provide important implications for policymakers who have the responsibility of allocating resources to improve children’s health. Since it is reasonable to expected parents with higher education and better health to have a greater probability of having healthier children, a program such as the BFP might contribute to the health and education of vulnerable individuals during gestational age and childhood. The BFP can also be considered a way to decrease inequality of opportunity in the long run.

## Supplementary Information

**Additional file 1.**

**Additional file 2.**

**Additional file 3: Table S1.** Descriptive statistics of our database compared to the Brazilian population. **Table S2.** Descriptive Statistics – Antenatal case visits. **Table S3.** Results from the logistic multilevel regression estimates including antenatal care visits. **Table S4.** Sensitivity analysis of missing at random (MAR) assumption for Low Birth Weight. **Table S5.** Sensitivity analysis of missing at random (MAR) assumption for Very Low Birth Weight. **Table S6.** Sensitivity analysis of missing at random (MAR) assumption for Preterm Birth. **Table S7.** Sensitivity analysis of missing at random (MAR) assumption for Very Preterm Birth. **Table S8.** Sensitivity analysis of missing at random (MAR) assumption for Congenital Malformation.

## Data Availability

The data that support the findings of this study are available from Center for Data and Knowledge Integration for Health (CIDACS) but restrictions apply to the availability of these data, which were used under license for the current study, and so are not publicly available. Data are however available from the authors upon reasonable request and with permission of CIDACS.

## Abbreviations

BF: *Bolsa Familia*
CFM: Congenital malformations
ICD: International Classification of Diseases
LBW: Low birth weight
MAR: Missing at random
PBF: *Bolsa Familia* Program
PTB: Preterm birth
VLBW: Very low birth weight
VPTB: Very preterm birth

## References

[1] Halliday T, Mazumder B, Wong A (2020). The intergenerational transmission of health in the United States: a latent variables analysis. Health Econ.

[2] Willson AE, Shuey KM (2019). A longitudinal analysis of the intergenerational transmission of health inequality. J Gerontol B.

[3] Bhalotra S, Rawlings S (2013). Gradients of the intergenerational transmission of health in developing countries. Rev Econ Stat.

[4] Schulkind L (2017). Getting a sporting chance: title IX and the intergenerational transmission of health. Health Econ.

[5] Bevis LE, Villa K. Intergenerational transmission of maternal health: evidence from Cebu, the Philippines. J Hum Resour. 2020.

[6] Fiszbein A (2009). Conditional cash transfers : reducing present and future poverty, in world bank policy research report.

[7] Medeiros M, Britto T, Soares FV. Targeted cash transfer programmes in Brazil: BPC and the Bolsa Familia. Working Paper number 46. International Poverty Center., 2008.

[8] Mourão L, Macedo de Jesus A. Bolsa Família (Family Grant) programme: an analysis of Brazilian income transfer programme. Field actions science reports, 2011. Special Issue 3.

[9] Lagarde M, Haines A, Palmer N (2007). Conditional cash transfers for improving uptake of health interventions in low- and middle-income countries a systematic review. JAMA.

[10] Gertler P (2004). Do conditional cash transfers improve child health? Evidence from PROGRESA’s control randomized experiment. Am Econ Rev.

[11] Rawlings LB (2005). A new approach to social assistance: Latin America’s experience with conditional cash transfer programmes. Int Soc Secur Rev.

[12] Baird S (2010). The short-term impacts of a schooling conditional cash transfer program on the sexual behavior of young women. Health Econ.

[13] Rasella D (2013). Effect of a conditional cash transfer programme on childhood mortality: a nationwide analysis of Brazilian municipalities. Lancet.

[14] Kakwani N, Veras Soares F, Son HH. Conditional cash transfers in African countries. 2005, International Policy Centre for Inclusive Growth.

[15] Hoffman R. Transferências de renda e desigualdade no Brasil (1995-2011), in Programa Bolsa Familia. Uma década de Inclusão e cidadania, T. Campello and M.C. Neri, Editors. 2013, Ipea - Instituto de Pesquisa Econômica Aplicada.

[16] Caixa Economica Federam: http://www.caixa.gov.br/programas-sociais/bolsa-familia/Paginas/default.aspx . (Accessed 8 July 2020).

[17] Paiva LH, Cotta TC, Barrientos A, Compton MEP, Hart T (2019). Brazil's Bolsa Família Programme, in great policy successes.

[18] Santos LMVV (2010). Bolsa familia programme: economic and social impacts under the perspective of the capabilities approach, in Bien 2010 Brazil.

[19] Jannuzzi P, Pinto AR, Campello T, Neri MC (2013). Bolsa Famılia e Seus Impactos nas Condiçoes de Vida da Populaçao Brasileira, in Programa Bolsa Familia. Uma década de Inclusão e cidadania.

[20] de Brauw A (2015). The impact of Bolsa Família on schooling. World Dev.

[21] Machado AF et al. Assessment of the implications of the Bolsa Família Programme for the decent work agenda. Working Papers 85, International Policy Centre for Inclusive Growth, 2011.

[22] Hellmann AG. How does Bolsa Familia work? Best practices in the implementation of conditional cash transfer programs in Latin America and the Caribbean. IDB Techn Note, 2015. 856.

[23] Duflo E (2000). Child health and household resources in South Africa: evidence from the old age pension program. Am Econ Rev.

[24] Habenschus MIAT, LG Scorzafave. Qual o efeito de longo prazo do Bolsa Família? Determinantes da chance de saída do programa ao longo de 10 anos, in LEPES - Laboratorio de Estudos e Pesquisas em Economia Social. 2019.

[25] Blumenshine P (2010). Socioeconomic disparities in adverse birth outcomes: a systematic review. Am J Prev Med.

[26] Vettore MV (2010). Housing conditions as a social determinant of low birthweight and preterm low birthweight. Rev Saude Publica.

[27] Matin A (2008). Maternal socioeconomic and nutritional determinants of low birth weight in urban area of Bangladesh. J Dhaka Medi Coll.

[28] Taywade ML, Pisudde PM (2017). Study of sociodemographic determinants of low birth weight in Wardha district, India. Clin Epidemiol Global Health.

[29] Assefa N, Berhane Y, Worku A (2012). Wealth status, mid upper arm circumference (MUAC) and Antenatal Care (ANC) are determinants for low birth weight in Kersa, Ethiopia. PLoS ONE.

[30] Sebayang SK (2012). Determinants of low birthweight, small-for-gestational-age and preterm birth in Lombok, Indonesia: analyses of the birthweight cohort of the SUMMIT trial. Tropical Med Int Health.

[31] Mahumud RA, Sultana M, Sarker AR (2017). Distribution and determinants of low birth weight in developing countries. J Prev Med Public Health.

[32] Kader M, Perera NKPP (2014). Socio-economic and nutritional determinants of low birth weight in India. N Am J Med Sci.

[33] Glassman A (2013). Impact of conditional cash transfers on maternal and newborn health. J Health Popul Nutr.

[34] Barber SL, Gertler PJ (2008). The impact of Mexico’s conditional cash transfer programme, Oportunidades, on birthweight. Tropical Med Int Health.

[35] Bonomo TP. Impacts of Bolsa Família program on infant health, in FGV EPGE - Dissertações, Mestrado em Economia 2018: Rio de Janeiro.

[36] Dolatian M (2014). Relationship between structural and intermediary determinants of health and preterm delivery. J Reprod Infertil.

[37] Leal MdC (2016). Prevalence and risk factors related to preterm birth in Brazil. Reprod Health.

[38] Fontoura FC, Cardoso MVLML (2014). Association between congenital malformation and neonatal and maternal variables in neonatal units of a Northeast Brazilian city. Texto Contexto Enfermagem.

[39] Basso O, Olsen J, Christensen K (1999). Recurrence risk of congenital anomalies--the impact of paternal, social, and environmental factors: a population-based study in Denmark. Am J Epidemiol.

[40] Vrijheid M (2000). Socioeconomic inequalities in risk of congenital anomaly. Arch Dis Child.

[41] Wasserman CR (1998). Socioeconomic status, neighborhood social conditions, and neural tube defects. Am J Public Health.

[42] Morales-Suárez Varela MM (2009). Socio-occupational status and congenital anomalies. Eur J Pub Health.

[43] Yu D (2014). Maternal socioeconomic status and the risk of congenital heart defects in offspring: a meta-analysis of 33 studies. PLoS ONE.

[44] Yang J (2007). Socioeconomic status in relation to selected birth defects in a large multicentered US case-control study. Am J Epidemiol.

[45] Rosano A, Del Bufalo E, Burgio A (2008). Socioeconomic status and risk of congenital malformations. Epidemiol Prev.

[46] Belbasis L (2016). Birth weight in relation to health and disease in later life: an umbrella review of systematic reviews and meta-analyses. BMC Med.

[47] Eide MG (2007). Associations of birth size, gestational age, and adult size with intellectual performance: evidence from a cohort of Norwegian men. Pediatr Res.

[48] Kormos CE (2013). Low birth weight and intelligence in adolescence and early adulthood: a meta-analysis. J Public Health.

[49] Flensborg-Madsen T, Mortensen EL (2017). Birth weight and intelligence in young adulthood and midlife. Pediatrics.

[50] Zerbeto AB, Cortelo FM, Filho ÉBC (2015). Association between gestational age and birth weight on the language development of Brazilian children: a systematic review. J Pediatr.

[51] Silveira VM, Horta BL (2008). Birth weight and metabolic syndrome in adults: meta-analysis. Rev Saude Publica.

[52] Yarmolinsky J (2016). Sex-specific associations of low birth weight with adult-onset diabetes and measures of glucose homeostasis: Brazilian longitudinal study of adult health. Sci Rep.

[53] Siewert-Delle A, Ljungman S (1998). The impact of birth weight and gestational age on blood pressure in adult life: a population-based study of 49-year-old men. Am J Hypertens.

[54] Boyle EM (2012). Effects of gestational age at birth on health outcomes at 3 and 5 years of age: population based cohort study. BMJ.

[55] Wolke D, Johnson S, Mendonça M (2019). The life course consequences of very preterm birth. Ann Rev Dev Psychol.

[56] Cronin FM (2016). Gestational age at birth and ‘body-mind’ health at 5 years of age: a population based cohort study. PLoS ONE.

[57] Wray J, Maynard L (2005). Living with congenital or acquired cardiac disease in childhood: maternal perceptions of the impact on the child and family. Cardiol Young.

[58] Latal B (2009). Psychological adjustment and quality of life in children and adolescents following open-heart surgery for congenital heart disease: a systematic review. BMC Pediatr.

[59] Wieser S (2018). How much does the treatment of each major disease cost? A decomposition of Swiss National Health Accounts. Eur J Health Econ.

[60] Brazilian Ministry of Citizenship, Cadastro Unico: https://aplicacoes.mds.gov.br/sagi/portal/index.php?grupo=212. (Accessed Aug 2019).

[61] Brazilian Ministry of Health, DATASUS. http://www2.datasus.gov.br/DATASUS/index.php?area=0901&item=1&acao=28&pad=31655. (Accessed Aug 2019).

[62] Pita R (2018). On the accuracy and scalability of probabilistic data linkage over the Brazilian 114 million cohort. IEEE J Biomed Health Inf.

[63] Barbosa GCG (2020). CIDACS-RL: a novel indexing search and scoring-based record linkage system for huge datasets with high accuracy and scalability. BMC Med Inform Decis Mak.

[64] Zhang K (2020). Expenditure and financial burden for stomach cancer diagnosis and treatment in china: a multicenter study. Front Public Health.

[65] Najafi F (2016). Productivity costs and years of potential life lost associated with five leading causes of death: Evidence from Iran (2006-2010). Med J Islam Repub Iran.

[66] Leidl R, Reitmeir P (2011). A value set for the EQ-5D based on experienced health states. PharmacoEconomics.

[67] World Health Organization, ICD-10 Version 2019: https://icd.who.int/browse10/2019/en. (Accessed Aug 2019).

[68] Carpenter, Kenward MG (2007). Missing data in randomised controlled trials: a practical guide.

[69] White IR, Royston P, Wood AM (2011). Multiple imputation using chained equations: Issues and guidance for practice. Stat Med.

[70] Rutstein S, Johnson K (2004). The DHS wealth index. DHS comparative reports.

[71] Araújo BFd, Tanaka ACdA (2007). Fatores de risco associados ao nascimento de recém-nascidos de muito baixo peso em uma população de baixa renda. Cadern Saúd Públ.

[72] Lindelow M (2018). Evaluating the impact of Bolsa familia on health outcomes: a critical review, in Bolsa Família 15 Anos (2003-2018).

[73] Paes-Sousa R, Santos LMP. Measuring the impact of Bolsa Familia program based on data from health and nutrition days (brazil). United Nations Food and Agriculture Organization (FAO), 2009. Hunger-Free Latin America and the Caribbean Initiative.

[74] Rasella D et al. Efeitos do programa Bolsa Família sobre a mortalidade em crianças: uma análise nos municípios brasileiros, in Programa Bolsa Familia. Uma década de Inclusão e cidadania, T. Campello and M.C. Neri, Editors. 2013, Ipea - Instituto de Pesquisa Econômica Aplicada. p. 247-396.

[75] Carniel EDF (2008). Determinantes do baixo peso ao nascer a partir das Declarações de Nascidos Vivos. Rev Br Epidemiol.

[76] Mesquita Costa G (2014). Determinantes d baixo peso ao nascer a partir das declarações de nascidos vivos / Low birth weigh determinants present in the statement of live birth. Cienc Enferm.

[77] Guimarães EAdA, Velásquez-Meléndez G (2002). Determinantes do baixo peso ao nascer a partir do Sistema de Informação sobre Nascidos Vivos em Itaúna, Minas Gerais. Rev Br Saúde Materno Infantil.

[78] Barbas DS (2009). Determinantes do peso insuficiente e do baixo peso ao nascer na cidade do Rio de Janeiro, Brasil, 2001. Epidemiol Serviços Saúde.

[79] Hunter BM (2017). The effects of cash transfers and vouchers on the use and quality of maternity care services: a systematic review. PLoS ONE.

[80] Celhay P et al. Paying patients for prenatal care: the effect of a small cash transfer on stillbirths and survival. 2017, IDB Working Paper Series.

[81] Santos DB (2017). The impact of the bolsa família program on the duration of formal employment of people with low income. Rev Admin Pública.

[82] de Sousa ALG (2016). Fatores determinantes para o nascimento de neonatos de baixo peso internados pelo método canguru. Rev Interdiscip.

[83] Pedraza DF, et al. Baixo peso ao nascer no Brasil: revisão sistemática de estudos baseados no sistema de informações sobre nascidos vivos. Pediatr Mod. 2014;50(2).

[84] Hernández-Díaz S, Schisterman EF, Hernán MA (2006). The birth weight “paradox” uncovered?. Am J Epidemiol.

[85] Juárez S, Ploubidis GB, Clarke L (2014). Revisiting the ‘low birth weight paradox’ using a model-based definition. Gac Sanit.

[86] Silva AAM (2010). The epidemiologic paradox of low birth weight in Brazil. Rev Saude Publica.

[87] Defo BK, Partin M (1993). Determinants of low birthweight: a comparative study. J Biosoc Sci.

[88] Tsimbos C, Verropoulou G (2011). Demographic and socioeconomic determinants of low birth weight and preterm births among natives and immigrants in Greece: an analysis using nationwide vital registration micro-data. J Biosoc Sci.

[89] Silva AMR (2009). Fatores de risco para nascimentos pré-termo em Londrina, Paraná, Brasil. Cadern Saúd Públ.

[90] Sharma SR (2015). Low birth weight at term and its determinants in a tertiary hospital of nepal: a case-control study. PLoS ONE.

[91] Silveira MF (2010). Determinants of preterm birth: Pelotas, Rio Grande do Sul State, Brazil, 2004 birth cohort. Cadern Saúd Públ.

