# Supplementary material for: The intergenerational relationship between conditional cash transfers and newborn health
Source: BMC Public Health. 2022 Jan 30;22:201. doi: 10.1186/s12889-022-12565-7 (PMC8801108; doi:10.1186/s12889-022-12565-7)
Supplement: Supplementary file 1 — Additional file 1. [file 12889_2022_12565_MOESM1_ESM.docx]

import delimited /home/Dados/Coorte_GCE/GCE_05/DB_ORIGINAL/02_gce_5_20190523_baseline_pop100_v2_base_sinasc_v2.csv

*(134 vars, 31,331,817 obs)

*************************** IMPORTANT DATES **************************

*People entered in the program by year

gen dt_entrada_coorte2=date(dt_entrada_coorte,"YMD")

format dt_entrada_coorte2 %td

gen dt_entrada_coorte2_year=year(dt_entrada_coorte2)

*People born each year

gen dtnasc_cidacs2=date(dtnasc_cidacs, "YMD")

format dtnasc_cidacs2 %td

gen dtnasc_cidacs2_year=year(dtnasc_cidacs2)

gen age= 2015-dtnasc_cidacs2_year

* Dates of beginning and end of receving Bolsa Familia

foreach i in dt_inicio_receb_fam dt_final_receb_fam {

gen `i'2=date(`i',"YMD")

format `i'2 %td

gen `i'2_year=year(`i'2)

}

gen diff_date=dt_final_receb_fam2-dt_inicio_receb_fam2

gen diferenca=qtd_dias_receb_fam-diff_date

************************ CONTROL VARIABLES ***************************

*Marital status

recode cd_estado_civil 0=. 88=. 3=4 5=3

label define cd_estado_civil_label 1 "Single" 2 "Married" ///

3 "Widowed" 4 "Divorced"

label var cd_estado_civil "Marital status"

label values cd_estado_civil cd_estado_civil_label

*Female*

recode sexo_cidacs 0=. //Female (2) or male (1)

rename sexo_cidacs Female

recode Female 2=1 1=0

label define Female 1 "Female" 0 "Male"

*Rural:

recode cod_local_domic_fam_eq 0=. //Rural (2) or urban (1)

rename cod_local_domic_fam_eq Rural

recode Rural 2=1 1=0

label define Rural 1 "Rural" 0 "Urban"

*City:

tostring municres_cidacs, gen(municres_cidacs_string) //Generate a string variable for the municipal code

rename municres_cidacs city

*Education:

recode escolaridade_eq 0=. 99=. 77=0

label define escolaridade_label 0 "No education" 1 "Literacy" ///

2 "Until 5th grade" 3 "Until 9th grade" 4 "Secondary or more"

label values escolaridade escolaridade_label

label var escolaridade "Education"

*Race:

recode racacor_cidacs 0=.

label define raca_label 1 "White" 2 "Black" 3 "Yellow" 4 "Brown" ///

5 "Indigenous"

label values raca raca_label

label var raca "Race/color"

*Place of birth

recode locnasc 0=. 99=.

label define locnasc_label 1 "Hospital" 2 "Other health establishment" /// 3 "At home" 4 "Other"

label values locnasc locnasc_label

label var locnasc "Place of birth"

*Pregnancy

recode gravidez 88=. 0=.

label define gravidez_label 1 "Single" 2 "Double" 3 "Triple"

label values gravidez gravidez_label

label var gravidez "Pregnancy"

*Ante-natal visits (use "consultas" and not "consprenat")

recode consultas 0=. 88=. 99=.

label define consultas_label 1 "0-3" 3 "4-6" 4 "More than 6"

label values consultas consultas_label

label var consultas "Antenatal visits"

*Previous fetal loss

replace qtdfilmort=. if qtdfilmort>10 & qtdfilmort!=.

gen deadchild=0

replace deadchild=1 if qtdfilmor>0

replace deadchild=. if qtdfilmor==.

rename deadchild Previous_fetal_loss

*First pregnancy

gen first_pregn=0

replace first_pregn=1 if qtdgestant<1

replace first_pregn=. if qtdgestant==.

*************** VARIABLES FOR WEALTH INDEX ***************************

*** Wealth index *** SMALLER VALUES INDICATE BETTER SOCIO ECONOMIC STATUS

recode cod_iluminacao_domic_fam_eq 0=. 6=. //Light in the house

recode cod_abaste_agua_domic_fam_eq 0=. //Water provision

recode cod_banheiro_domic_fam 0=. //Is there a bathroom at home

recode cod_calcamento_domic_fam 0=. //Is there asphalt in the street

recode cod_destino_lixo_domic_fam_eq 0=. //Garbage

recode cod_escoa_sanitario_domic_fam_eq 0=. //Sewage (Fogna)

recode cod_especie_domic_fam_eq 0=. 4=. //Kind of house (permanent building, slum, etc). KEEP IT OUT.

recode cod_material_domic_fam_eq 0=. //Material of house building

replace qtd_comodos_domic_fam_eq=. if qtd_comodos_domic_fam_eq>10 & qtd_comodos_domic_fam_eq!=. //Number of rooms

replace qtd_comodos_domic_fam_eq=6 if qtd_comodos_domic_fam_eq>5 & qtd_comodos_domic_fam_eq!=.

recode qtd_comodos_domic_fam_eq 0=. 1=6 2=5 3=4 4=3 5=2 6=1 //Number of families in the house

replace qtd_familias_domic_fam=. if qtd_familias_domic_fam>10 & qtd_familias_domic_fam!=.

replace qtd_familias_domic_fam=6 if qtd_familias_domic_fam>6 & qtd_familias_domic_fam!=.

recode cd_situacao_domicilio 6=1 7=. 0=. //House property, rent or other

recode cd_tipo_domicilio 0=. //House property (house, apt, room, etc.)

recode cd_tratamento_agua 0=. //Treatment of water

recode cod_agua_canalizada_fam 0=. //Water comes from the tubes or not

recode cod_material_piso_fam 0=. 7=. 6=1 5=2 4=3 3=4 2=5 1=6 //Floor material

*cd_construcao: don`t use because there are less observations than cod_material_domic_fam_eq

*cd_escoamento_sanitario: don`t use because there are less observations than cod_escoa_sanitario_domic_fam_eq

*qtd_comodos_dormitorio_fam: don`t use because there are less observations than qtd_comodos_domic_fam_eq

drop cd_construcao cd_escoamento_sanitario qtd_comodos_dormitorio_fam

* NB; The variables with a decent level of observations are; cod_iluminacao_domic_fam_eq, cod_abaste_agua_domic_fam_eq, cod_destino_lixo_domic_fam_eq ,

*cod_escoa_sanitario_domic_fam_eq, cod_especie_domic_fam_eq, cod_material_domic_fam_eq, (qtd_comodos_domic_fam_eq)

*PCA for socio-economic status variables

*Gather variables for inclusion in factor analysis

factor cod_iluminacao_domic_fam_eq cod_abaste_agua_domic_fam_eq cod_banheiro_domic_fam cod_calcamento_domic_fam cod_destino_lixo_domic_fam_eq ///

cod_escoa_sanitario_domic_fam_eq cod_especie_domic_fam_eq cod_material_domic_fam_eq qtd_comodos_domic_fam_eq qtd_familias_domic_fam ///

cd_situacao_domicilio cd_tipo_domicilio cd_tratamento_agua cod_agua_canalizada_fam cod_material_piso_fam, pcf

*factor analysis with a reduced number of variables (including only variables with few missing)

factor cod_iluminacao_domic_fam_eq cod_abaste_agua_domic_fam_eq cod_banheiro_domic_fam cod_destino_lixo_domic_fam_eq ///

cod_escoa_sanitario_domic_fam_eq cod_especie_domic_fam_eq cod_material_domic_fam_eq, pcf

estat kmo

*The Kaiser-Meyer-Olkin measure of sampling adequacy varies b/w 0 and 1. Thevalues closer to 1 are the better. A value of 0.6 is ///

*a suggested minimumacceptable value. IN our example we get 0.7 which is fine.

*Raw sum forcing for missing

egen sum_wealth =rowtotal (cod_iluminacao_domic_fam_eq cod_abaste_agua_domic_fam_eq cod_destino_lixo_domic_fam_eq ///

cod_escoa_sanitario_domic_fam_eq cod_especie_domic_fam_eq cod_material_domic_fam_eq qtd_comodos_domic_fam_eq ///

cd_situacao_domicilio cd_tipo_domicilio cd_tratamento_agua), missing

*Raw sum without including the missing

egen sum_wealth_nomissing =rowtotal (cod_iluminacao_domic_fam_eq cod_abaste_agua_domic_fam_eq cod_destino_lixo_domic_fam_eq ///

cod_escoa_sanitario_domic_fam_eq cod_especie_domic_fam_eq cod_material_domic_fam_eq qtd_comodos_domic_fam_eq ///

cd_situacao_domicilio cd_tipo_domicilio cd_tratamento_agua)

drop score1 ses_5cat

* From the factor analysis we create a predicted score for each household based on the factor

predict score1

* Use the predicted scores to create 5 categories of wealth from poorest to the least poor

egen ses_5cat=cut(score1), group(5)

label define ses5_label 0 "Poorest" 1 "Poor" 2 "Middle" 3 "Rich" 4 "Richest"

label values ses_5cat ses5_label

* Test to see if everything is correct

tabstat score1, by(ses_5cat) statistics(mean) col(var)

**********************************************************************

************************* DEPENDENT VARIABLES ************************

recode gestacao 88=. 99=. 0=. //Weeks of pregnancy

label define gestacao_labels 1 "Less than 22" 2 "22-27" 3 "28-31" 4 "32-36" 5 "37-41" 6 "42 or more"

label values gestacao gestacao_labels

label var gestacao "Weeks of pregnancy"

*Diseases:

gen sick1=substr(codanomal, 1,4)

*64,150 values

gen sick2=substr(codanomal, 5, 4)

*2216 values

gen sick3=substr(codanomal, 9, 4)

*497 values

gen sick4=substr(codanomal, 13, 4)

*153 values

gen sick5=substr(codanomal, 17, 4)

*52 values

gen sick6=substr(codanomal, 21, 4)

*0 values

drop sick6

*Identify Congenital malformation****

*CID Q****

gen s1=substr(codanomal, 1,1)

gen s2=substr(codanomal, 5,1)

gen s3=substr(codanomal, 9,1)

gen s4=substr(codanomal, 13,1)

gen s5=substr(codanomal, 17,1)

*Congenital malformation:

gen CMF=0

replace CMF=1 if s1=="Q"

replace CMF=1 if s2=="Q"

replace CMF=1 if s3=="Q"

replace CMF=1 if s4=="Q"

replace CMF=1 if s5=="Q"

replace peso=. if peso>7600 & peso!=.

*Low birth weight:

gen LBW=0

replace LBW=1 if peso<2500 & peso!=.

*Very low birth weigh:

gen VLBW=0

replace VLBW=1 if peso<1500 & peso!=.

* Premature:

gen PTB=0

replace PTB=1 if gestacao<5 & gestacao!=.

* Very premature:

gen VPTB=0

replace VPTB=1 if gestacao<3 & gestacao!=.

save "DataCleaned.dta"

**********************************************************************

******************** Merge mother and child **************************

use "DataCleaned.dta", clear

/* ********************DEFINITION OF VARIABLES**********************************

id_cidacs_pop100_v2: cidacs id, this code is unique for every individual

cod_familiar_eq: family code

ind_mae_sinasc_cidacs==1: 0 if not mother, 1 if mother

id_cidacs_mae_sinasc: mother`s cidacs id

*/

**********************************************************************

*********************** MOTHER DATASET *******************************

keep if ind_mae_sinasc_cidacs==1

keep id_cidacs_pop100_v2 age city cd_estado_civil_label escolaridade_eq racacor_cidacs id_cidacs_mae_sinasc

foreach var of varlist id_cidacs_pop100_v2 age city cd_estado_civil_label escolaridade_eq racacor_cidacs id_cidacs_mae_sinasc {

rename `var' `var'_M

}

rename id_cidacs_pop100_v2 mother

save "MotherDataset.dta", replace

**********************************************************************

*MERGE CHILD AND MOTHER:

use "DataCleaned.dta", clear

* Merge dataset children and mothers together

rename id_cidacs_mae_sinasc mother

br mother

merge m:1 mother using "MotherDataset.dta"

keep if _merge==3

drop _merge

*Tot observations: 16,448,931

*we have 1,110 identified grandmothers

save "MotherANDChilDataset.dta"

**********************************************************************

*MERGE CHILD, MOTHERS AND CITIES:

use "MotherANDChilDataset.dta", clear

merge m:1 city_M using "/home/Dados/Coorte_GCE/GCE_05/Shared/ExternalDatasetToBeMERGED23oct2019.dta"

keep if _merge==3

drop _merge

save "MotherChilANDCitiesDataset.dta"

********************** ADDITIONAL VARIABLES **************************

use "MotherChilANDCitiesDataset.dta", clear

*Mother age

gen age_at_birth=.

replace age_at_birth = age_M - age

gen maternity_age2=.

replace maternity_age2=0 if age_at_birth>=10 & age_at_birth<20 & age_at_birth!=.

replace maternity_age2=1 if age_at_birth>=20 & age_at_birth<30 & age_at_birth!=.

replace maternity_age2=2 if age_at_birth>=30 & age_at_birth<40 & age_at_birth!=.

replace maternity_age2=3 if age_at_birth>=40 & age_at_birth<50 & age_at_birth!=.

label define maternity_age2_label 0 "10-19" 1 "20-29" 2 "30-39" 3 "40 plus"

label values maternity_age2 maternity_age2_label

* Poverty reduction

gen pov_red=(p_pob_2000-p_pob_2010)/100

*BF uptake

gen BF_uptake=0

replace BF_uptake=1 if dtnasc_cidacs2 > dt_inicio_receb_fam & dtnasc_cidacs2!=. & dt_inicio_receb_fam!=.

*Prob early exposure

egen bf_mother_incidence=cut(ind_bf_mother), group(5)

label define bf_mother_incidence_label 0 "Very low" 1 "Low" 2 "Middle" 3 "High" 4 "Very High"

label values bf_mother_incidence bf_mother_incidence_label

save "FinalDataset.dta"

************************** REGRESSIONS *******************************

use "FinalDataset.dta", clear

*Regressions:

global model_1 BF_uptake i.bf_mother_incidence pov_red gdp_r_pc i.wealth_index ///

i.escolaridade_eq2_M i.maternity_age2 i.estcivmae female i.racacor_cidacs rural ///

i.gravidez first_pregn Previous_fetal_loss i.locnasc i.year

global model_2 BF_uptake i.bf_mother_incidence pov_red gdp_r_pc i.wealth_index ///

i.escolaridade_eq2_M i.maternity_age2 i.estcivmae female i.racacor_cidacs rural ///

i.gravidez first_pregn Previous_fetal_loss i.locnasc i.visits i.year

melogit LBW $model_1 || city: , or

melogit LBW $model_2 || city: , or

melogit VLBW $model_1 || city: , or

melogit VLBW $model_2 || city: , or

melogit PTB $model_1 || city: , or

melogit PTB $model_2 || city: , or

melogit VPTB $model_1 || city: , or

melogit VPTB $model_2 || city: , or

melogit CMF $model_1 || city: , or

melogit CMF $model_2 || city: , or

************************ MULTIPLE IMPUTATION *************************

use "FinalDataset.dta", clear

sum LBW VLBW PTB VPTB CMF pov_red gdp_r_pc BF_uptake bf_mother_incidence female ///

racacor_cidacs wealth_index locnasc city estcivmae escolaridade_eq2_M visits ///

maternity_age2 first_pregn Previous_fetal_loss gravidez rural year

keep LBW VLBW PTB VPTB CMF pov_red gdp_r_pc BF_uptake bf_mother_incidence female ///

racacor_cidacs wealth_index locnasc city estcivmae escolaridade_eq2_M visits ///

maternity_age2 first_pregn Previous_fetal_loss gravidez rural year

mi set flong

mi register imputed l LBW VLBW PTB VPTB CMF pov_red gdp_r_pc BF_uptake bf_mother_incidence female ///

racacor_cidacs wealth_index locnasc city estcivmae escolaridade_eq2_M visits ///

maternity_age2 first_pregn Previous_fetal_loss gravidez rural year

set matsize 11000, permanently

mi impute chained (logit) LBW VLBW PTB VPTB CMF female first_pregn ///

Previous_fetal_loss rural BF_uptake (ologit) bf_mother_incidence wealth_index ///

escolaridade_eq2_M visits (mlogit) locnasc racacor_cidacs estcivmae maternity_age2 ///

gravidez year (regress) pov_red gdp_r_pc city, add(5) rseed(1234) augment

save "DatasetMI.dta"

*Regressions:

use "DatasetMI.dta", clear

global model_1 BF_uptake i.bf_mother_incidence pov_red gdp_r_pc i.wealth_index ///

i.escolaridade_eq2_M i.maternity_age2 i.estcivmae female i.racacor_cidacs rural ///

i.gravidez first_pregn Previous_fetal_loss i.locnasc i.year

global model_2 BF_uptake i.bf_mother_incidence pov_red gdp_r_pc i.wealth_index ///

i.escolaridade_eq2_M i.maternity_age2 i.estcivmae female i.racacor_cidacs rural ///

i.gravidez first_pregn Previous_fetal_loss i.locnasc i.visits i.year

xi: mi estimate, or cmdok: melogit LBW $model_1 || city:

xi: mi estimate, or cmdok: melogit LBW $model_2 || city:

xi: mi estimate, or cmdok: melogit VLBW $model_1 || city:

xi: mi estimate, or cmdok: melogit VLBW $model_2 || city:

xi: mi estimate, or cmdok: melogit PTB $model_1 || city:

xi: mi estimate, or cmdok: melogit PTB $model_2 || city:

xi: mi estimate, or cmdok: melogit VPTB $model_1 || city:

xi: mi estimate, or cmdok: melogit VPTB $model_2 || city:

xi: mi estimate, or cmdok: melogit CMF $model_1 || city:

xi: mi estimate, or cmdok: melogit CMF $model_2 || city:

/*
