# Supplementary material for: The intergenerational relationship between conditional cash transfers and newborn health
Source: BMC Public Health. 2022 Jan 30;22:201. doi: 10.1186/s12889-022-12565-7 (PMC8801108; doi:10.1186/s12889-022-12565-7)
Supplement: Supplementary file 2 — Additional file 2. [file 12889_2022_12565_MOESM2_ESM.docx]

*Step 1: create missings dataset

use "DatasetMI.dta", clear

misstable sum BF_uptake i.bf_mother_incidence pov_red gdp_r_pc i.wealth_index ///

i.escolaridade_eq2_M i.maternity_age2 i.estcivmae female i.racacor_cidacs rural ///

i.gravidez first_pregn Previous_fetal_loss i.locnasc i.year LBW VLBW PTB VPTB CMF if _mi_m==0, gen(miss_)

save "DatasetMI_robust.dta"

keep _mi_id _mi_m miss_*

keep if _mi_m==0

save "Missings.dta"

*Step 2: merge with MI dataset

use "DatasetMI.dta", clear

merge m:1 _mi_id using "Missings.dta"

***************************** Scenarios ******************************

/*

Scenario 1: we assumed that people with missing education were 20% more educated than the observed

Scenario 2: we assumed that people with missing education were 20% less educated than the observed

Scenario 3: we assumed that people with missing wealth were 20% poorer than the observed

Scenario 4: we assumed that people with missing wealth were 20% richer than the observed

Scenario 5: we assumed that mothers with the variable “previous fetal loss” missing, had a previous fetal loss

Scenario 6: we assumed that mothers with the variable “previous fetal loss” missing, did not have a previous fetal loss

Scenario 7: we assumed that people with missing observations were 20% less educated, 20% poorer than the observed and had a previous fetal loss

Scenario 8: we assumed that people with missing observations were 20% more educated, 20% richer than the observed and did not have a previous fetal loss

*/

*Education+1:

gen edu2=escolaridade_eq2_M+miss_escolaridade_eq2_M

recode edu2 5=4

*Education-1:

gen edu3=escolaridade_eq2_M-miss_escolaridade_eq2_M

recode edu3 -1=0

*Wealth +1:

gen wealth2=wealth_index+miss_wealth_index

recode wealth2 5=4

*Wealth -1:

gen wealth3=wealth_index-miss_wealth_index

recode wealth3 -1=0

*Fetal lost missing=1:

gen Previous_fetal_loss2=Previous_fetal_loss

replace Previous_fetal_loss2=1 if miss_Previous_fetal_loss==1

*Fetal lost missing=0:

gen Previous_fetal_loss3=Previous_fetal_loss

replace Previous_fetal_loss3=0 if miss_Previous_fetal_loss==1

*labels:

label define edu2_label 0 "No education" 1 "Literacy" 2 "Until 5th" 3 "Until 9th" 4 "Secondary or more"

label values edu2 edu2_label

label define edu3_label 0 "No education" 1 "Literacy" 2 "Until 5th" 3 "Until 9th" 4 "Secondary or more"

label values edu3 edu3_label

label define wealth2_label 0 "Richest" 1 "Rich" 2 "Middle" 3 "Poor" 4 "Poorest"

label values wealth2 wealth2_label

label define wealth3_label 0 "Richest" 1 "Rich" 2 "Middle" 3 "Poor" 4 "Poorest"

label values wealth3 wealth3_label

global main_analysis BF_uptake i.bf_mother_incidence pov_red gdp_r_pc i.wealth_index ///

i.escolaridade_eq2_M i.maternity_age2 i.estcivmae female i.racacor_cidacs rural ///

i.gravidez first_pregn Previous_fetal_loss i.locnasc i.year

global Scenario_1 BF_uptake i.bf_mother_incidence pov_red gdp_r_pc i.wealth_index ///

i.edu2 i.maternity_age2 i.estcivmae female i.racacor_cidacs rural ///

i.gravidez first_pregn Previous_fetal_loss i.locnasc i.year

global Scenario_2 BF_uptake i.bf_mother_incidence pov_red gdp_r_pc i.wealth_index ///

i.edu3 i.maternity_age2 i.estcivmae female i.racacor_cidacs rural ///

i.gravidez first_pregn Previous_fetal_loss i.locnasc i.year

global Scenario_3 BF_uptake i.bf_mother_incidence pov_red gdp_r_pc i.wealth2 ///

i.escolaridade_eq2_M i.maternity_age2 i.estcivmae female i.racacor_cidacs rural ///

i.gravidez first_pregn Previous_fetal_loss i.locnasc i.year

global Scenario_4 BF_uptake i.bf_mother_incidence pov_red gdp_r_pc i.wealth3 ///

i.escolaridade_eq2_M i.maternity_age2 i.estcivmae female i.racacor_cidacs rural ///

i.gravidez first_pregn Previous_fetal_loss i.locnasc i.year

global Scenario_5 BF_uptake i.bf_mother_incidence pov_red gdp_r_pc i.wealth_index ///

i.escolaridade_eq2_M i.maternity_age2 i.estcivmae female i.racacor_cidacs rural ///

i.gravidez first_pregn Previous_fetal_loss2 i.locnasc i.year

global Scenario_6 BF_uptake i.bf_mother_incidence pov_red gdp_r_pc i.wealth_index ///

i.escolaridade_eq2_M i.maternity_age2 i.estcivmae female i.racacor_cidacs rural ///

i.gravidez first_pregn Previous_fetal_loss3 i.locnasc i.year

global Scenario_7 BF_uptake i.bf_mother_incidence pov_red gdp_r_pc i.wealth2 ///

i.edu3 i.maternity_age2 i.estcivmae female i.racacor_cidacs rural ///

i.gravidez first_pregn Previous_fetal_loss2 i.locnasc i.year

global Scenario_8 BF_uptake i.bf_mother_incidence pov_red gdp_r_pc i.wealth3 ///

i.edu2 i.maternity_age2 i.estcivmae female i.racacor_cidacs rural ///

i.gravidez first_pregn Previous_fetal_loss3 i.locnasc i.year

xi: mi estimate, or cmdok: melogit LBW $main_analysis || city:

xi: mi estimate, or cmdok: melogit LBW $Scenario_1 || city:

xi: mi estimate, or cmdok: melogit LBW $Scenario_2 || city:

xi: mi estimate, or cmdok: melogit LBW $Scenario_3 || city:

xi: mi estimate, or cmdok: melogit LBW $Scenario_4 || city:

xi: mi estimate, or cmdok: melogit LBW $Scenario_5 || city:

xi: mi estimate, or cmdok: melogit LBW $Scenario_6 || city:

xi: mi estimate, or cmdok: melogit LBW $Scenario_7 || city:

xi: mi estimate, or cmdok: melogit LBW $Scenario_8 || city:

xi: mi estimate, or cmdok: melogit VLBW $main_analysis || city:

xi: mi estimate, or cmdok: melogit VLBW $Scenario_1 || city:

xi: mi estimate, or cmdok: melogit VLBW $Scenario_2 || city:

xi: mi estimate, or cmdok: melogit VLBW $Scenario_3 || city:

xi: mi estimate, or cmdok: melogit VLBW $Scenario_4 || city:

xi: mi estimate, or cmdok: melogit VLBW $Scenario_5 || city:

xi: mi estimate, or cmdok: melogit VLBW $Scenario_6 || city:

xi: mi estimate, or cmdok: melogit VLBW $Scenario_7 || city:

xi: mi estimate, or cmdok: melogit VLBW $Scenario_8 || city:

xi: mi estimate, or cmdok: melogit PTB $main_analysis || city:

xi: mi estimate, or cmdok: melogit PTB $Scenario_1 || city:

xi: mi estimate, or cmdok: melogit PTB $Scenario_2 || city:

xi: mi estimate, or cmdok: melogit PTB $Scenario_3 || city:

xi: mi estimate, or cmdok: melogit PTB $Scenario_4 || city:

xi: mi estimate, or cmdok: melogit PTB $Scenario_5 || city:

xi: mi estimate, or cmdok: melogit PTB $Scenario_6 || city:

xi: mi estimate, or cmdok: melogit PTB $Scenario_7 || city:

xi: mi estimate, or cmdok: melogit PTB $Scenario_8 || city:

xi: mi estimate, or cmdok: melogit VPTB $main_analysis || city:

xi: mi estimate, or cmdok: melogit VPTB $Scenario_1 || city:

xi: mi estimate, or cmdok: melogit VPTB $Scenario_2 || city:

xi: mi estimate, or cmdok: melogit VPTB $Scenario_3 || city:

xi: mi estimate, or cmdok: melogit VPTB $Scenario_4 || city:

xi: mi estimate, or cmdok: melogit VPTB $Scenario_5 || city:

xi: mi estimate, or cmdok: melogit VPTB $Scenario_6 || city:

xi: mi estimate, or cmdok: melogit VPTB $Scenario_7 || city:

xi: mi estimate, or cmdok: melogit VPTB $Scenario_8 || city:

xi: mi estimate, or cmdok: melogit CMF $main_analysis || city:

xi: mi estimate, or cmdok: melogit CMF $Scenario_1 || city:

xi: mi estimate, or cmdok: melogit CMF $Scenario_2 || city:

xi: mi estimate, or cmdok: melogit CMF $Scenario_3 || city:

xi: mi estimate, or cmdok: melogit CMF $Scenario_4 || city:

xi: mi estimate, or cmdok: melogit CMF $Scenario_5 || city:

xi: mi estimate, or cmdok: melogit CMF $Scenario_6 || city:

xi: mi estimate, or cmdok: melogit CMF $Scenario_7 || city:

xi: mi estimate, or cmdok: melogit CMF $Scenario_8 || city:
