# Supplementary material for: The intergenerational relationship between conditional cash transfers and newborn health
Source: BMC Public Health. 2022 Jan 30;22:201. doi: 10.1186/s12889-022-12565-7 (PMC8801108; doi:10.1186/s12889-022-12565-7)
Supplement: Supplementary file 3 — Additional file 3: Table S1. Descriptive statistics of our database compared to the Brazilian population. Table S2. Descriptive Statistics – Antenatal case visits. Table S3. Results from the logistic multilevel regression estimates including antenatal care visits. Table S4. Sensitivity analysis of missing at random (MAR) assumption for Low Birth Weight. Table S5. Sensitivity analysis of missing at random (MAR) assumption for Very Low Birth Weight. Table S6. Sensitivity analysis of missing at random (MAR) assumption for Preterm Birth. Table S7. Sensitivity analysis of missing at random (MAR) assumption for Very Preterm Birth. Table S8. Sensitivity analysis of missing at random (MAR) assumption for Congenital Malformation. [file 12889_2022_12565_MOESM3_ESM.docx]

Supplementary material

**Table S1: Descriptive statistics of our database compared to the Brazilian population**

| **Variable** | **Our sample  (CIDACS)** | | **Brazilian population (SINASC)** | |
| --- | --- | --- | --- | --- |
|  | **N. observations** | **Mean** | **N. observations** | **Mean** |
| **Child health outcomes** |  |  |  |  |
| LBW | 5,242,209 | 0.073 | 14,709,270 | 0.085 |
| VLBW | 5,242,209 | 0.007 | 14,709,270 | 0.013 |
| PTB | 5,019,754 | 0.108 | 14,194,415 | 0.114 |
| VPTB | 5,019,754 | 0.002 | 14,194,415 | 0.005 |
| CFM | 5,246,673 | 0.006 | 14,719,903 | 0.008 |
| **Social indicators** |  |  |  |  |
| Mother age group |  |  |  |  |
| 10-19 | 5,245,195 | 0.242 | 14,717,771 | 0.190 |
| 20-29 | 5,245,195 | 0.498 | 14,717,771 | 0.501 |
| 29-39 | 5,245,195 | 0.245 | 14,717,771 | 0.286 |
| 40 plus | 5,245,195 | 0.024 | 14,717,771 | 0.024 |
| Marital status |  |  |  |  |
| Single | 5,170,896 | 0.484 | 14,537,912 | 0.429 |
| Married | 5,170,896 | 0.506 | 14,537,912 | 0.559 |
| Widow | 5,170,896 | 0.002 | 14,537,912 | 0.002 |
| Divorced | 5,170,896 | 0.008 | 14,537,912 | 0.010 |
| Female child | 5,246,673 | 0.489 | 14,717,272 | 0.488 |
| Race/color of the child |  |  |  |  |
| White | 5,246,673 | 0.328 | 14,116,725 | 0.390 |
| Black | 5,246,673 | 0.035 | 14,116,725 | 0.051 |
| Yellow | 5,246,673 | 0.006 | 14,116,725 | 0.004 |
| Brown | 5,246,673 | 0.622 | 14,116,725 | 0.547 |
| Indigenous | 5,246,673 | 0.009 | 14,116,725 | 0.008 |
| Rural | 5,246,118 | 0.216 | - | - |
| **Health indicators** |  |  |  |  |
| Pregnancy |  |  |  |  |
| Single | 5,235,314 | 0.986 | 14,692,723 | 0.980 |
| Double | 5,235,314 | 0.014 | 14,692,723 | 0.020 |
| Triple | 5,235,314 | 0.000 | 14,692,723 | 0.001 |
| First pregnancy | 4,503,804 | 0.306 | - | - |
| Previous fetal loss | 4,655,849 | 0.195 | - | - |
| Place of birth |  |  |  |  |
| Hospital | 5,245,547 | 0.980 | 14,718,991 | 0.983 |
| Other health establishment | 5,245,547 | 0.009 | 14,718,991 | 0.008 |
| Home | 5,245,547 | 0.009 | 14,718,991 | 0.008 |
| Other | 5,245,547 | 0.002 | 14,718,991 | 0.002 |
| N | 5,246,874 |  | 14,719,903 |  |

Note: LBW = low birth weight; VLBW = very low birth weight, PTB = pre-term birth, VPTB = very pre-term birth, CFM = congenital malformations.

**Table S2: Descriptive Statistics – Antenatal case visits**

| **N. of antenatal care visits** | **Our sample  (CIDACS)** | | | **Brazilian population (SINASC)** | |
| --- | --- | --- | --- | --- | --- |
|  | **N. observations** | | **Mean** | **N. observations** | **Mean** |
| 0-3 | 5,206,184 | 0.108 | | 14,589,739 | 0.097 |
| 4-6 | 5,206,184 | 0.316 | | 14,589,739 | 0.264 |
| More than 6 | 5,206,184 | 0.575 | | 14,589,739 | 0.639 |

**Table S3: Results from the logistic multilevel regression estimates including antenatal care visits**

|  | LBW | VLBW | PTB | VPTB | CMF |
| --- | --- | --- | --- | --- | --- |
| **BF Exposure** |  |  |  |  |  |
| BF uptake | 0.951*** | 0.904*** | 1.003 | 0.989 | 1.014 |
|  | [0.942-0.960] | [0.879-0.931] | [0.996-1.010] | [0.944-1.037] | [0.985-1.045] |
| Prob early exposure |  |  |  |  |  |
| Q1 (Lowest) | (reference) | (reference) | (reference) | (reference) | (reference) |
| Q2 | 0.898*** | 0.800*** | 0.908*** | 0.834** | 0.808*** |
|  | [0.868-0.930] | [0.745-0.860] | [0.880-0.937] | [0.746-0.933] | [0.745-0.877] |
| Q3 | 0.791*** | 0.652*** | 0.863*** | 0.755*** | 0.712*** |
|  | [0.764-0.81993] | [0.604-0.703] | [0.836-0.890] | [0.670-0.851] | [0.653-0.776] |
| Q4 | 0.660*** | 0.548*** | 0.812*** | 0.704*** | 0.678*** |
|  | [0.635-0.686] | [0.504-0.597] | [0.785-0.841] | [0.618-0.803] | [0.616-0.747] |
| Q5 (Highest) | 0.614*** | 0.573*** | 0.832*** | 0.778*** | 0.611*** |
|  | [0.590-0.639] | [0.525-0.626] | [0.803-0.862] | [0.678-0.891] | [0.553-0.676] |
| **Economic indicators** |  |  |  |  |  |
| Poverty_reduction | 1.003*** | 1.004** | 1.001 | 1.004 | 1.002 |
|  | [1.002-1.005] | [1.001-1.007] | [1.000-1.002] | [1.000-1.009] | [0.999-1.006] |
| GDP_per_capita | 1 | 1.001* | 1.000* | 1 | 1 |
|  | [1.000-1.001] | [1.000-1.002] | [1.000-1.001] | [0.999-1.001] | [0.999-1.001] |
| Wealth Index |  |  |  |  |  |
| Q1 (Richest) | (reference) | (reference) | (reference) | (reference) | (reference) |
| Q2 | 0.997 | 0.922*** | 0.985** | 0.915* | 0.978 |
|  | [0.985-1.010] | [0.889-0.956] | [0.975-0.996] | [0.855-0.980] | [0.942-1.016] |
| Q3 | 0.986* | 0.905*** | 0.966*** | 0.887*** | 0.955* |
|  | [0.973-0.998] | [0.867-0.944] | [0.954-0.977] | [0.833-0.943] | [0.916-0.995] |
| Q4 | 0.981** | 0.851*** | 0.957*** | 0.818*** | 0.976 |
|  | [0.969-0.995] | [0.818-0.885] | [0.947-0.968] | [0.758-0.883] | [0.938-1.017] |
| Q5 (Poorest) | 0.961*** | 0.778*** | 0.938*** | 0.766*** | 0.943* |
|  | [0.947-0.975] | [0.745-0.812] | [0.927-0.950] | [0.703-0.834] | [0.900--0.988] |
| **Social indicators** |  |  |  |  |  |
| Mother education |  |  |  |  |  |
| No education | (reference) | (reference) | (reference) | (reference) | (reference) |
| Literacy | 0.989 | 1.154 | 1.002 | 1.216 | 0.903 |
|  | [0.943-1.037] | [0.988-1.349] | [0.968-1.037] | [0.999-1.480] | [0.760-1.073] |
| Until 5th year | 0.978* | 1.027 | 0.987 | 1.01 | 0.983 |
|  | [0.961-0.996] | [0.973-1.084] | [0.968-1.006] | [0.920-1.108] | [0.925-1.045] |
| Until 9th year | 0.943*** | 1.109*** | 0.926*** | 0.989 | 0.98 |
|  | [0.926-0.960] | [1.049-1.172] | [0.910-0.942] | [0.904-1.082] | [0.924-1.040] |
| Secondary or more | 0.929*** | 1.285*** | 0.927*** | 1.097* | 0.981 |
|  | [0.910-0.947] | [1.210-1.365] | [0.910-0.945] | [1.003-1.200] | [0.922-1.044] |
| Mother age group |  |  |  |  |  |
| 10-19 | (reference) | (reference) | (reference) | (reference) | (reference) |
| 20-29 | 0.958*** | 1.056** | 0.830*** | 0.790*** | 1.004 |
|  | [0.948-0.968] | [1.022- 1.091] | [0.823-0.838] | [0.751-0.831] | [0.970-1.039] |
| 29-39 | 1.191*** | 1.555*** | 0.959*** | 0.905** | 1.133*** |
|  | [1.176-1.206] | [1.497-1.615] | [0.948-0.969] | [0.850-0.963] | [1.089-1.179] |
| 40 plus | 1.634*** | 2.024*** | 1.219*** | 1.111 | 1.735*** |
|  | [1.600-1.670] | [1.901-2.153] | [1.197-1.242] | [0.995-1.241] | [1.627-1.851] |
| Marital status |  |  |  |  |  |
| Single | (reference) | (reference) | (reference) | (reference) | (reference) |
| Married | 0.996 | 1.090*** | 1.041*** | 0.937-0.018** | 1.022 |
|  | [0.989-1.003] | [1.066-1.115] | [1.035-1.048] | [0.902-0.973] | [0.998-1.048] |
| Widow | 0.989 | 0.93 | 1.009 | 1.029 | 1.336* |
|  | [0.913-1.072] | [0.728-1.188] | [0.944-1.079] | [0.679-1.558] | [1.070-1.670] |
| Divorced | 1.053** | 1.133* | 1.097*** | 0.984 | 1.125* |
|  | [1.015-1.093] | [1.021-1.258] | [1.063-1.132] | [0.792-1.223] | [1.008-1.256] |
| Female child | 1.292*** | 1.168*** | 0.940*** | 1.041* | 0.730*** |
|  | [1.284-1.301] | [1.144-1.193] | [0.934-0.945] | [1.002-1.082] | [0.714-0.747] |
| Race/color of the child |  |  |  |  |  |
| White | (reference) | (reference) | (reference) | (reference) | (reference) |
| Black | 1.121*** | 1.032 | 1.046*** | 1.09 | 0.979 |
|  | [1.101-1.142] | [0.977-1.089] | [1.030-1.063] | [0.992-1.198] | [0.922-1.040] |
| Yellow | 1.048 | 1.054 | 1.013 | 0.861 | 1.020926 |
|  | [1.001-1.098] | [0.920-1.208] | [0.975-1.053] | [0.667-1.111] | [0.879-1.185] |
| Brown | 1.038*** | 0.964** | 1.019 | 1.017 | 0.931*** |
|  | [1.030-1.047] | [0.940-0.989] | [1.012-1.027] | [0.971-1.065] | [0.907-0.956] |
| Indigenous | 0.846*** | 0.549*** | 1.142*** | 0.602*** | 0.89 |
|  | [0.806-0.888] | [0.468-0.644] | [1.104-1.182] | [0.472-0.768] | [0.760-1.044] |
| Rural | 0.945*** | 0.963* | 0.945*** | 0.954 | 1.013 |
|  | [0.934-0.956] | [0.929-0.998] | [0.936-0.954] | [0.892-1.022] | [0.974-1.053] |
| **Health indicators** |  |  |  |  |  |
| Pregnancy |  |  |  |  |  |
| Single | (reference) | (reference) | (reference) | (reference) | (reference) |
| Double | 19.057*** | 9.735*** | 8.839*** | 5.679*** | 1.256*** |
|  | [18.765-19.354] | [9.413-10.068] | [8.701-8.979] | [5.291-6.095] | [1.157-1.363] |
| Triple | 68.548*** | 57.579*** | 33.761*** | 17.620*** [13.153-23.605] | 1.182 |
|  | [59.475-79.004] | [50.307-65.902] | [29.518-38.614] |  | [0.634-2.204] |
| First pregnancy | 1.805*** | 2.117*** | 1.202*** | 1.469*** | 1.245*** |
|  | [1.787-1.823] | [2.052-2.184] | [1.190-1.214] | [1.399-1.544] | [1.208-1.283] |
| Previous fetal loss | 1.425*** | 1.730*** | 1.169*** | 1.376*** | 1.159*** |
|  | [1.411-1.437] | [1.683-1.779] | [1.158-1.180] | [1.311-1.444] | [1.123-1.196] |
| Place of birth |  |  |  |  |  |
| Hospital | (reference) | (reference) | (reference) | (reference) | (reference) |
| Other health establishment | 0.658*** | 0.519*** | 0.847*** | 0.905 | 1.108 |
|  | [0.628-0.691] | [0.440-0.613] | [0.815-0.880] | [0.737-1.112] | [0.972-1.263] |
| Home | 1.303*** | 1.374*** | 1.373*** | 1.517*** | 0.812** |
|  | [1.258- 1.350] | [1.241-1.521] | [1.331-1.417] | [1.290-1.784] | [0.698-0.946] |
| Other | 2.714*** | 2.574*** | 1.796*** | 2.369*** | 1.384* |
|  | [2.545-2.894] | [2.194-3.020] | [1.699-1.899] | [1.847-3.038] | [1.076-1.779] |
| Antenatal visits |  |  |  |  |  |
| 0-3 | (reference) | (reference) | (reference) | (reference) | (reference) |
| 4-6 | 0.661*** | 0.465*** | 0.685*** | 0.310*** | 0.907*** |
|  | [0.654-0.668] | [0.454-0.477] | [0.679-0.691] | [0.298-0.323] | [0.872-0.944] |
| More than 6 | 0.317*** | 0.114*** | 0.289*** | 0.061*** | 0.866*** |
|  | [0.313-0.320] | [0.111-0.118] | [0.286-0.291] | [0.058-0.064] | [0.833-0.901] |
| N | 5246874 | 5246874 | 5246874 | 5246874 | 5246874 |

Note: LBW = low birth weight; VLBW = very low birth weight, PTB = pre-term birth, VPTB = very pre-term birth, CFM = congenital malformations. Year fixed effects included in the estimate. Results presented in Odds Ratios; 95% conﬁdence intervals in brackets. *P<0.05; **P<0.01; ***P<0.001.

**S2 SENSITIVITY ANALYSIS FOR THE MAR ASSUMPTION OF THE MULTIPLE IMPUTATION**

We challenged the assumption of missing values being Missing at Random (MAR) and tested a Missing Not at Random (MNAR) assumption. We followed the “Pattern-mixture models” approach that tests the hypothesis of different distributions between the missing and observed data [1]. In particular, the distribution of a variable is supposed to be a combination of the distribution of the observed and missing values (‘pattern-mixture’). For example, mothers with missing data might have a 10% lower LBW children than those observed. To obtain the imputed data under MNAR, we rescaled each MAR-imputed value, i.e. multiplied imputed values by a constant factor, as in Leurent et al [1]. We ran 6 scenarios where we changed one parameter at a time and 2 scenarios where we change all parameters at the same time. Tables S4-S8 show the results obtained for each health outcome under each scenario.

The sensitivity analysis confirmed the consistency of results in case of departures from the MAR assumption. Results were overall very stable.

***Scenarios***

Scenario 1: we assumed that people with missing education were 20% more educated than the observed

Scenario 2: we assumed that people with missing education were 20% less educated than the observed

Scenario 3: we assumed that people with missing wealth were 20% poorer than the observed

Scenario 4: we assumed that people with missing wealth were 20% richer than the observed

Scenario 5: we assumed that mothers with the variable “previous fetal loss” missing, had a previous fetal loss

Scenario 6: we assumed that mothers with the variable “previous fetal loss” missing, did not have a previous fetal loss

Scenario 7: we assumed that people with missing observations were 20% less educated, 20% poorer than the observed and had a previous fetal loss

Scenario 8: we assumed that people with missing observations were 20% more educated, 20% richer than the observed and did not have a previous fetal loss

**Table S4: Sensitivity analysis of missing at random (MAR) assumption for Low Birth Weight**

|  | Main analysis | Scenario 1 | Scenario 2 | Scenario 3 | Scenario 4 |
| --- | --- | --- | --- | --- | --- |
| **BF Exposure** |  |  |  |  |  |
| BF uptake | 0.932*** | 0.933*** | 0.934*** | 0.932*** | 0.933*** |
|  | [0.924-0.941] | [0.925-0.941] | [0.926-0.942] | [0.924-0.940] | [0.925-0.940] |
| Prob early exposure |  |  |  |  |  |
| Q1 (Lowest) | (reference) | (reference) | (reference) | (reference) | (reference) |
| Q2 | 0.919*** | 0.910*** | 0.910*** | 0.910*** | 0.910*** |
|  | [0.894-0.944] | [0.886-0.935] | [0.886-0.935] | [0.886-0.934] | [0.886-0.934] |
| Q3 | 0.831*** | 0.818*** | 0.818*** | 0.816*** | 0.817*** |
|  | [0.807-0.854] | [0.795-0.840] | [0.795-0.840] | [0.794-0.839] | [0.795-0.840] |
| Q4 | 0.741*** | 0.728*** | 0.728*** | 0.726*** | 0.727*** |
|  | [0.719-0.765] | [0.706-0.750] | [0.706-0.751] | [0.704-0.749] | [0.705-0.749] |
| Q5 (Highest) | 0.690*** | 0.678*** | 0.678*** | 0.676*** | 0.676*** |
|  | [0.668-0.713] | [0.657-0.700] | [0.657-0.700] | [0.655-0.697] | [0.655-0.698] |
|  | Main analysis | Scenario 5 | Scenario 6 | Scenario 7 | Scenario 8 |
| **BF Exposure** |  |  |  |  |  |
| BF uptake | 0.932*** | 0.935*** | 0.931*** | 0.935*** | 0.933*** |
|  | [0.924-0.941] | [0.927-0.943] | [0.923-0.939] | [0.927-0.943] | [0.925-0.941] |
| Prob early exposure |  |  |  |  |  |
| Q1 (Lowest) | (reference) | (reference) | (reference) | (reference) | (reference) |
| Q2 | 0.919*** | 0.909*** | 0.910*** | 0.910*** | 0.911*** |
|  | [0.894-0.944] | [0.885-0.934] | [0.886-0.935] | [0.886-0.934] | [0.887-0.935] |
| Q3 | 0.831*** | 0.815*** | 0.818*** | 0.815*** | 0.819*** |
|  | [0.807-0.854] | [0.793-0.838] | [0.796-0.841] | [0.793-0.838] | [0.797-0.842] |
| Q4 | 0.741*** | 0.719*** | 0.730*** | 0.720*** | 0.732*** |
|  | [0.719-0.765] | [0.698-0.742] | [0.708-0.753] | [0.698-0.742] | [0.710-0.754] |
| Q5 (Highest) | 0.690*** | 0.664*** | 0.682*** | 0.665*** | 0.683*** |
|  | [0.668-0.713] | [0.643-0.686] | [0.660-0.704] | [0.644-0.687] | [0.662-0.705] |

Notes: “Prob early exposure” indicates the probability of social protection exposure during mother’s childhood. Year fixed effects included in the estimate. Results presented in odds ratios; 95% conﬁdence intervals in brackets. *P<0.05; **P<0.01; ***P<0.001.

**Table S5: Sensitivity analysis of missing at random (MAR) assumption for Very Low Birth Weight**

|  | Main analysis | Scenario 1 | Scenario 2 | Scenario 3 | Scenario 4 |
| --- | --- | --- | --- | --- | --- |
| **BF Exposure** |  |  |  |  |  |
| BF uptake | 0.872*** | 0.867*** | 0.869*** | 0.868*** | 0.868*** |
|  | [0.848-0.897] | [0.844-0.891] | [0.845-0.892] | [0.845-0.892] | [0.845-0.892] |
| Prob early exposure |  |  |  |  |  |
| Q1 (Lowest) | (reference) | (reference) | (reference) | (reference) | (reference) |
| Q2 | 0.849*** | 0.837*** | 0.837*** | 0.838*** | 0.836*** |
|  | [0.799-0.903] | [0.787-0.890] | [0.787-0.890] | [0.788-0.891] | [0.786-0.889] |
| Q3 | 0.738*** | 0.719*** | 0.720*** | 0.721*** | 0.716*** |
|  | [0.691-0.790] | [0.673-0.768] | [0.673-0.769] | [0.675-0.771] | [0.670-0.765] |
| Q4 | 0.695*** | 0.669*** | 0.671*** | 0.673*** | 0.665*** |
|  | [0.645-0.749] | [0.621-0.722] | [0.622-0.723] | [0.624-0.726] | [0.617-0.717] |
| Q5 (Highest) | 0.707*** | 0.678*** | 0.680*** | 0.683*** | 0.674*** |
|  | [0.653-0.765] | [0.627-0.733] | [0.628-0.735] | [0.631-0.739] | [0.623-0.729] |
|  | Main analysis | Scenario 5 | Scenario 6 | Scenario 7 | Scenario 8 |
| **BF Exposure** |  |  |  |  |  |
| BF uptake | 0.872*** | 0.872*** | 0.866*** | 0.871*** | 0.865*** |
|  | [0.848-0.897] | [0.849-0.896] | [0.842-0.890] | [0.848-0.895] | [0.842-0.889] |
| Prob early exposure |  |  |  |  |  |
| Q1 (Lowest) | (reference) | (reference) | (reference) | (reference) | (reference) |
| Q2 | 0.849*** | 0.835*** | 0.838*** | 0.836*** | 0.837*** |
|  | [0.799-0.903] | [0.786-0.888] | [0.788-0.892] | [0.786-0.888] | [0.787-0.891] |
| Q3 | 0.738*** | 0.716*** | 0.727*** | 0.717*** | 0.719*** |
|  | [0.691-0.790] | [0.670-0.765] | [0.676-0.773] | [0.671-0.766] | [0.672-0.769] |
| Q4 | 0.695*** | 0.660*** | 0.676*** | 0.662*** | 0.670*** |
|  | [0.645-0.749] | [0.612-0.711] | [0.627-0.729] | [0.614-0.713] | [0.621-0.723] |
| Q5 (Highest) | 0.707*** | 0.660*** | 0.688*** | 0.662*** | 0.682*** |
|  | [0.653-0.765] | [0.610-0.714] | [0.636-0.744] | [0.612-0.715] | [0.630-0.738] |

Notes: “Prob early exposure” indicates the probability of social protection exposure during mother’s childhood. Year fixed effects included in the estimate. Results presented in odds ratios; 95% conﬁdence intervals in brackets. *P<0.05; **P<0.01; ***P<0.001.

**Table S6: Sensitivity analysis of missing at random (MAR) assumption for Preterm Birth**

|  | Main analysis | Scenario 1 | Scenario 2 | Scenario 3 | Scenario 4 |
| --- | --- | --- | --- | --- | --- |
| **BF Exposure** |  |  |  |  |  |
| BF uptake | 0.979*** | 0.989** | 0.990** | 0.988*** | 0.988** |
|  | [0.971-0.986] | [0.982-0.995] | [0.984-0.997] | [0.981-0.995] | [0.982-0.995] |
| Prob early exposure |  |  |  |  |  |
| Q1 (Lowest) | (reference) | (reference) | (reference) | (reference) | (reference) |
| Q2 | 0.936*** | 0.932*** | 0.932*** | 0.932*** | 0.932*** |
|  | [0.910-0.962] | [0.907-0.958] | [0.907-0.957] | [0.907-0.957] | [0.907-0.957] |
| Q3 | 0.922*** | 0.914*** | 0.914*** | 0.912*** | 0.914*** |
|  | [0.897-0.949] | [0.889-0.940] | [0.889-0.940] | [0.888-0.938] | [0.889-0.939] |
| Q4 | 0.932*** | 0.921*** | 0.921*** | 0.918*** | 0.920*** |
|  | [0.904-0.961] | [0.893-0.949] | [0.893-0.949] | [0.890-0.946] | [0.892-0.948] |
| Q5 (Highest) | 0.946*** | 0.932*** | 0.932*** | 0.928*** | 0.930*** |
|  | [0.916-0.976] | [0.904-0.962] | [0.903-0.961] | [0.900-0.957] | [0.901-0.959] |
|  | Main analysis | Scenario 5 | Scenario 6 | Scenario 7 | Scenario 8 |
| **BF Exposure** |  |  |  |  |  |
| BF uptake | 0.979*** | 0.988** | 0.987*** | 0.989** | 0.990** |
|  | [0.971-0.986] | [0.982-0.995] | [0.980-0.994] | [0.982-0.996] | [0.983-0.997] |
| Prob early exposure |  |  |  |  |  |
| Q1 (Lowest) | (reference) | (reference) | (reference) | (reference) | (reference) |
| Q2 | 0.936*** | 0.932*** | 0.932*** | 0.932*** | 0.932*** |
|  | [0.910-0.962] | [0.907-0.957] | [0.901-0.957] | [0.907-0.957] | [0.907-0.958] |
| Q3 | 0.922*** | 0.913*** | 0.914*** | 0.913*** | 0.916*** |
|  | [0.897-0.949] | [0.888-0.938] | [0.889-0.939] | [0.888-0.938] | [0.891-0.941] |
| Q4 | 0.932*** | 0.916*** | 0.921*** | 0.916*** | 0.923*** |
|  | [0.904-0.961] | [0.889-0.944] | [0.893-0.949] | [0.889-0.944] | [0.896-0.951] |
| Q5 (Highest) | 0.946*** | 0.924*** | 0.932*** | 0.925*** | 0.935*** |
|  | [0.916-0.976] | [0.896-0.953] | [0.904-0.962] | [0.897-0.954] | [0.907-0.964] |

Notes: “Prob early exposure” indicates the probability of social protection exposure during mother’s childhood. Year fixed effects included in the estimate. Results presented in odds ratios; 95% conﬁdence intervals in brackets. *P<0.05; **P<0.01; ***P<0.001.

**Table S7: Sensitivity analysis of missing at random (MAR) assumption for Very Preterm Birth**

|  | Main analysis | Scenario 1 | Scenario 2 | Scenario 3 | Scenario 4 |
| --- | --- | --- | --- | --- | --- |
| **BF Exposure** |  |  |  |  |  |
| BF uptake | 0.930** | 0.924** | 0.925** | 0.923** | 0.924** |
|  | [0.887-0.975] | [0.882-0.968] | [0.883-0.969] | [0.881-0.967] | [0.882-0.968] |
| Prob early exposure |  |  |  |  |  |
| Q1 (Lowest) | (reference) | (reference) | (reference) | (reference) | (reference) |
| Q2 | 0.892* | 0.885* | 0.884* | 0.885* | 0.884* |
|  | [0.807-0.985] | [0.800-0.977] | [0.800-0.977] | [0.801-0.978] | [0.800-0.977] |
| Q3 | 0.885* | 0.871* | 0.871* | 0.871* | 0.870* |
|  | [0.795-0.986] | [0.782-0.971] | [0.782-0.971] | [0.781-0.971] | [0.781-0.969] |
| Q4 | 0.92 | 0.890* | 0.899 | 0.899 | 0.896 |
|  | [0.814-1.034] | [0.798-1.014] | [0.798-1.014] | [0.797-1.013] | [0.795-1.010] |
| Q5 (Highest) | 0.956 | 0.937 | 0.936 | 0.935 | 0.931 |
|  | [0.843-1.084] | [0.826-1.062] | [0.826-1.061] | [0.824-1.061] | [0.821-1.056] |
|  | Main analysis | Scenario 5 | Scenario 6 | Scenario 7 | Scenario 8 |
| **BF Exposure** |  |  |  |  |  |
| BF uptake | 0.930** | 0.926** | 0.922** | 0.926** | 0.924** |
|  | [0.887-0.975] | [0.884-0.970] | [0.879-0.966] | [0.884-0.970] | [0.882-0.968] |
| Prob early exposure |  |  |  |  |  |
| Q1 (Lowest) | (reference) | (reference) | (reference) | (reference) | (reference) |
| Q2 | 0.892* | 0.882* | 0.885* | 0.884* | 0.885* |
|  | [0.807-0.985] | [0.799-0.975] | [0.801-0.978] | [0.800-0.976] | [0.801-0.978] |
| Q3 | 0.885* | 0.867* | 0.873* | 0.868* | 0.873* |
|  | [0.795-0.986] | [0.778-0.966] | [0.783-0.973] | [0.778-0.967] | [0.784-0.972] |
| Q4 | 0.92 | 0.889 | 0.902 | 0.890 | 0.901 |
|  | [0.814-1.034] | [0.788-1.001] | [0.800-1.017] | [0.789-1.004] | [0.799-1.016] |
| Q5 (Highest) | 0.956 | 0.917 | 0.940 | 0.920 | 0.939 |
|  | [0.843-1.084] | [0.809-1.040] | [0.829-1.066] | [0.811-1.044] | [0.828-1.064] |

Notes: “Prob early exposure” indicates the probability of social protection exposure during mother’s childhood. Year fixed effects included in the estimate. Results presented in odds ratios; 95% conﬁdence intervals in brackets. *P<0.05; **P<0.01; ***P<0.001.

**Table S8: Sensitivity analysis of missing at random (MAR) assumption for Congenital Malformation**

|  | Main analysis | Scenario 1 | Scenario 2 | Scenario 3 | Scenario 4 |
| --- | --- | --- | --- | --- | --- |
| **BF Exposure** |  |  |  |  |  |
| BF uptake | 1.012 | 1.002 | 1.003 | 1.002 | 1.002 |
|  | [0.982-1.042] | [0.974-1.031] | [0.975-1.032] | [0.974-1.031] | [0.974-1.031] |
| Prob early exposure |  |  |  |  |  |
| Q1 (Lowest) | (reference) | (reference) | (reference) | (reference) | (reference) |
| Q2 | 0.810*** | 0.810*** | 0.810*** | 0.810*** | 0.809*** |
|  | [0.747-0.879] | [0.746-0.878] | [0.747-0.879] | [0.747-0.879] | [0.746-0.878] |
| Q3 | 0.716*** | 0.715*** | 0.716*** | 0.717*** | 0.713*** |
|  | [0.657-0.780] | [0.657-0.779] | [0.657-0.780] | [0.658-0.781] | [0.654-0.777] |
| Q4 | 0.686*** | 0.685*** | 0.686*** | 0.686*** | 0.682*** |
|  | [0.623-0.754] | [0.622-0.753] | [0.623-0.755] | [0.624-0.755] | [0.619-0.750] |
| Q5 (Highest) | 0.617*** | 0.616*** | 0.617*** | 0.617*** | 0.613*** |
|  | [0.558-0.681] | [0.557-0.680] | [0.558-0.682] | [0.559-0.682] | [0.555-0.678] |
|  | Main analysis | Scenario 5 | Scenario 6 | Scenario 7 | Scenario 8 |
| **BF Exposure** |  |  |  |  |  |
| BF uptake | 1.012 | 1.002 | 1.002 | 1.001 | 1.002 |
|  | [0.982-1.042] | [0.974-1.031] | [0.974-1.031] | [0.973-1.030] | [0.974-1.031] |
| Prob early exposure |  |  |  |  |  |
| Q1 (Lowest) | (reference) | (reference) | (reference) | (reference) | (reference) |
| Q2 | 0.810*** | 0.810*** | 0.810*** | 0.810*** | 0.809*** |
|  | [0.747-0.879] | [0.746-0.879] | [0.747-0.879] | [0.747-0.879] | [0.746-0.878] |
| Q3 | 0.716*** | 0.716*** | 0.716*** | 0.717*** | 0.714*** |
|  | [0.657-0.780] | [0.657-0.780] | [0.657-0.780] | [0.658-0.781] | [0.655-0.778] |
| Q4 | 0.686*** | 0.685*** | 0.686*** | 0.686*** | 0.684*** |
|  | [0.623-0.754] | [0.623-0.754] | [0.624-0.755] | [0.62-0.756] | [0.621-0.752] |
| Q5 (Highest) | 0.617*** | 0.616*** | 0.618*** | 0.617*** | 0.616*** |
|  | [0.558-0.681] | [0.557-0.681] | [0.560-0.683] | [0.558-0.682] | [0.558-0.681] |

Notes: “Prob early exposure” indicates the probability of social protection exposure during mother’s childhood. Year fixed effects included in the estimate. Results presented in odds ratios; 95% conﬁdence intervals in brackets. *P<0.05; **P<0.01; ***P<0.001.

**References**

1. Leurent, B., et al., *Sensitivity Analysis for Not-at-Random Missing Data in Trial-Based Cost-Effectiveness Analysis: A Tutorial.* PharmacoEconomics, 2018. **36**(8): p. 889-901.
